# Tissue-Specific Methylation Biosignatures for Monitoring Diseases: An In Silico Approach

**DOI:** 10.3390/ijms23062959

**Published:** 2022-03-09

**Authors:** Makrina Karaglani, Maria Panagopoulou, Ismini Baltsavia, Paraskevi Apalaki, Theodosis Theodosiou, Ioannis Iliopoulos, Ioannis Tsamardinos, Ekaterini Chatzaki

**Affiliations:** 1 Laboratory of Pharmacology, Medical School, Democritus University of Thrace, GR-68100 Alexandroupolis, Greece; mkaragla@med.duth.gr (M.K.); mpanagop@med.duth.gr (M.P.); bio2278apalaki@gmail.com (P.A.); theodosios.theodosiou@gmail.com (T.T.); 2 Department of Basic Sciences, School of Medicine, University of Crete, GR-71003 Heraklion, Greece; ibaltsavia@gmail.com (I.B.); iliop.john@gmail.com (I.I.); 3 JADBio Gnosis DA S.A., Science and Technology Park of Crete, GR-70013 Heraklion, Greece; tsamard@jadbio.com; 4 Department of Computer Science, University of Crete, GR-70013 Heraklion, Greece; 5 Institute of Applied and Computational Mathematics, Foundation for Research and Technology—Hellas, GR-70013 Heraklion, Greece; 6 Institute of Agri-Food and Life Sciences, Hellenic Mediterranean University Research Centre, GR-71410 Heraklion, Greece

**Keywords:** methylation, machine learning, microarrays, model, liquid biopsy, diabetes, breast cancer, osteoarthritis

## Abstract

Tissue-specific gene methylation events are key to the pathogenesis of several diseases and can be utilized for diagnosis and monitoring. Here, we established an in silico pipeline to analyze high-throughput methylome datasets to identify specific methylation fingerprints in three pathological entities of major burden, i.e., breast cancer (BrCa), osteoarthritis (OA) and diabetes mellitus (DM). Differential methylation analysis was conducted to compare tissues/cells related to the pathology and different types of healthy tissues, revealing Differentially Methylated Genes (DMGs). Highly performing and low feature number biosignatures were built with automated machine learning, including: (1) a five-gene biosignature discriminating BrCa tissue from healthy tissues (AUC 0.987 and precision 0.987), (2) three equivalent OA cartilage-specific biosignatures containing four genes each (AUC 0.978 and precision 0.986) and (3) a four-gene pancreatic β-cell-specific biosignature (AUC 0.984 and precision 0.995). Next, the BrCa biosignature was validated using an independent ccfDNA dataset showing an AUC and precision of 1.000, verifying the biosignature’s applicability in liquid biopsy. Functional and protein interaction prediction analysis revealed that most DMGs identified are involved in pathways known to be related to the studied diseases or pointed to new ones. Overall, our data-driven approach contributes to the maximum exploitation of high-throughput methylome readings, helping to establish specific disease profiles to be applied in clinical practice and to understand human pathology.

## 1. Introduction

DNA methylation is a well-characterized epigenetic mechanism participating in the regulation of gene expression, and is related to a variety of normal functions [1]. Aberrant gene methylation contributes to the pathophysiology of human diseases, such as cancer [2], autoimmune disorders [3] and diabetes [4]. The detection of alterations in DNA methylation, either on tissues or in liquid biopsies, has been involved in the initiation [5,6], progression [7,8,9] and response to the treatment of several diseases [10,11,12] and, thus, it is thought to hold valuable information for their clinical management. Genome-wide methylation analyses enable the study of a vast number of CpG sites and produce high-dimensional datasets that can be exploited for a deeper understanding of the contribution of methylation in human pathology. They also offer the opportunity to build specific biosignatures for personalized clinical solutions of clinical importance.

In parallel to the rapid accumulation of multiple high-throughput omics data, machine learning (ML) approaches have been introduced to enable their exploitation. ML uses a variety of algorithms that perform intelligent predictions and is highly applicable in biomarker discovery [13,14]. Specifically, ML applied to different type of omics datasets has been used for diagnosis or classification and prognosis in various cancers [15,16,17], neurological diseases [18], coronary artery disease [19], osteoarthritis [20] and diabetes [14]. However, developing an ML approach entails a lot of effort to select and configure the appropriate algorithm to process the data to learn from, among other things [21]. To this end, automated tools for ML (AutoML) have recently become available∙ they promise to democratize data analysis to non-experts, drastically increase productivity, improve the replicability of the statistical analysis, facilitate the interpretation of results, and shield against common methodological analysis pitfalls, such as overfitting [22]. Bioinformatic analysis combined with AutoML analysis of big omics datasets is able to extract knowledge and predictive models that can be used in personalized clinical decisions. To the best of our knowledge, only a few studies focusing on cancer have applied ML to methylation data analysis [15,16,17].

Cell-free DNA fragments circulate in the biological fluids of healthy and diseased individuals. The cellular release mechanisms of circulating cell-free DNA (ccfDNA) include apoptosis, necrosis and active release from viable cells. Recent studies have shown that multiple tissues contribute to the ccfDNA mixture of healthy individuals, while in disease, it is enriched also from pathological tissues [23,24]. ccfDNA fragments carry identical methylation footprints to their tissue of origin, serving as valuable liquid biopsy material, as they can dynamically mirror changes throughout the pathophysiological process [25]. Tracing in ccfDNA the methylation footprints of a tissue presents an unprecedented opportunity for early diagnosis and monitoring.

To tackle this major challenge in biomarker discovery, in the present study, we established an in silico pipeline based on high-throughput microarray methylation datasets to identify disease/tissue specific methylation fingerprints. Three pathological entities of major burden, i.e., one malignancy (breast cancer, BrCa), one inflammatory (osteoarthritis, OA) and one metabolic (diabetes) were selected as use cases in our approach. Instead of the comparison, adopted in most studies, of a pathological tissue to the respective healthy one (for example breast cancer tissue vs. normal breast), here we chose to compare methylomes from a tissue or cell type related to a specific pathology to the bulk of methylomes from other healthy tissues. Differential analysis revealed specific differentially methylated genes (DMGs) which were then subjected to functional analysis to unravel epigenetically regulated pathways in each pathology. Following this, AutoML technology, specially designed for analyzing high-dimensional biological datasets, was applied to build tissue-specific methylation biosignatures, validated also in ccfDNA. Selected features were additionally studied using a text mining bioinformatic tool to reveal their biological associations. Overall, our approach contributes to the maximum exploitation and knowledge mining of existing high-throughput methylome readings to establish specific disease profiles to be exploited in clinical practice and understand human pathology.

## 2. Results

### 2.1. Breast Cancer

#### 2.1.1. Differential Methylation Analysis Comparing BrCa and Healthy Tissues

**Figure 1:**
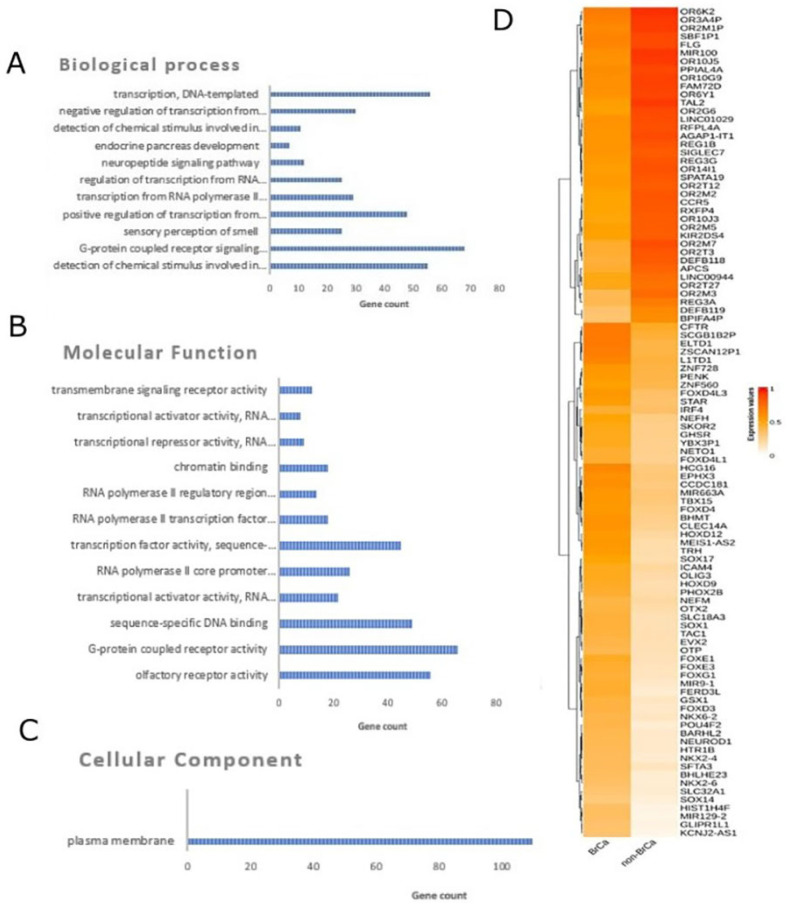
Differential methylation analysis comparing BrCa and healthy tissues. Gene ontology analysis of the top 400 DMGs in the aspects of (**A**) biological process, (**B**) cellular component and (**C**) molecular function analysis. (**D**) Heatmap plot of top 100 DMGs comparing BrCa and healthy tissues. Abbreviations: BrCa = breast cancer, DMGs = differentially methylated genes.

In order to identify differentially methylated genes in a comparison between BrCa tumors and healthy tissues, raw methylome data from 218 BrCa (primary and metastatic) tumors and 193 healthy tissues, including healthy breast, blood, liver, muscle, colon, gastric, lung and adipose (Supplementary Table S1), were subjected to analysis using RnBeads. In total, 19,248 DMGs (false discovery rate (FDR) < 0.05) emerged. Among those, 8820 were found to be hypomethylated, while 10,428 showed hypermethylation in BrCa in relation to healthy tissues. A heatmap visualization of DMGs is presented in Figure 1D. Further, DMGs were ranked based on FDR, and the 400 top-ranking genes were chosen for functional analysis. Of these 400 DMGs, 171 were hypomethylated and the remaining 229 were hypermethylated in BrCa in relation to healthy tissues. The complete list of the 400 top-ranking DMGs from the comparison between tissues is presented in Supplementary Table S2.

#### 2.1.2. Functional Analysis of DMGs Comparing BrCa and Healthy Tissues

Gene ontology analysis was carried out using the DAVID tool (Figure 1A–C). In molecular function analysis, the most enriched functions were G-protein-coupled receptor activity, sequence-specific DNA binding, transcriptional activator activity and RNA polymerase II core promoter proximal region sequence-specific binding. In biological process enrichment analysis, DMGs were found to participate mainly in G-protein-coupled receptor signaling pathways, the positive regulation of transcription from RNA polymerase II promoter, transcription from RNA and the polymerase II promoter regulation of transcription from RNA polymerase II promoter. Finally, cellular component analysis showed mainly a plasma membrane enrichment of the studied genes. Reactome analysis via ConsensusPathDB mainly revealed enrichment in sensory perception, the genetic transcription pathway, RNA polymerase II transcription and gene expression (Supplementary Figure S1). The protein–protein interaction (PPI) network of the 400 DMGs was visualized using the Cytoscape tool and is demonstrated in Supplementary Materials.

#### 2.1.3. BrCa-Specific Methylation Biosignature through AutoML

**Figure 2:**
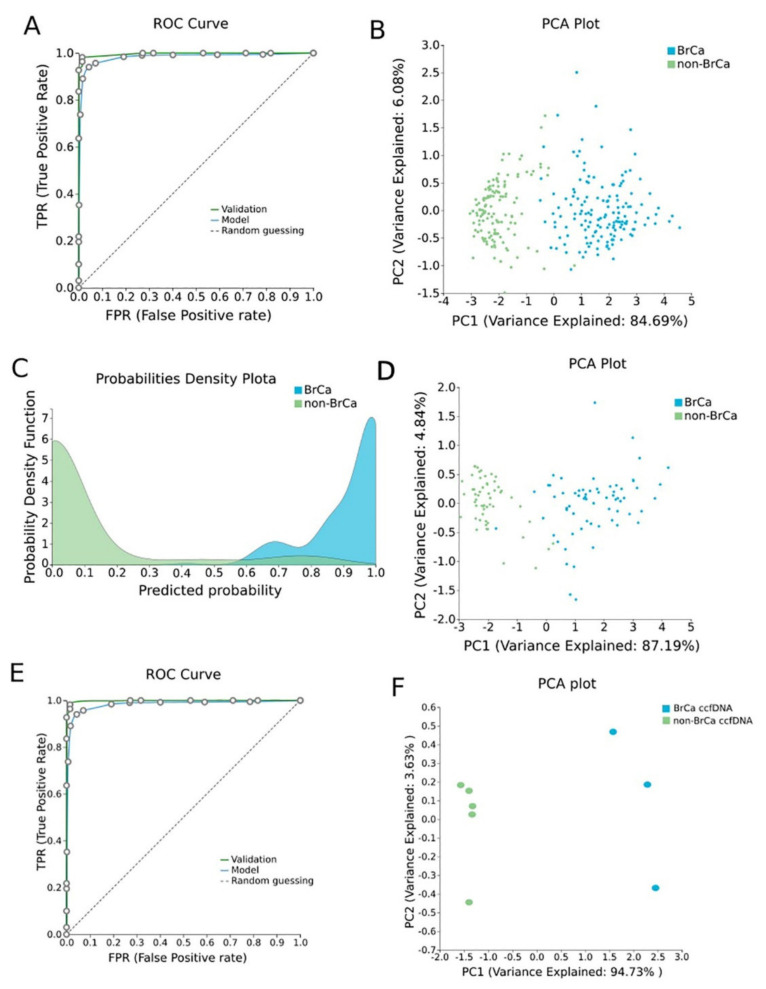
BrCa-specific methylation biosignature built using AutoML. (**A**) ROC curves of training (blue line) and validation (green line) models. (**B**) Supervised PCA plot (i.e., only considering the selected relevant biomarkers) presents separation between BrCa (blue) and healthy tissues (green) within the training group. (**C**) Out-of-sample probability density plot (i.e., probability predictions when samples were not used for training) depicts discrete distributions among studied classes of the training group. (**D**) PCA plot presents separation between BrCa (blue) and healthy tissues (green) within the validation group. (**E**) ROC curves of training (blue line) and external validation (green line) models and (**F**) PCA plot presents separation between BrCa ccfDNA (blue) and healthy ccfDNA (green) within the external validation group. Abbreviations: BrCa = breast cancer, ROC = receiver operating characteristic, PCA = principal component analysis.

β-values produced by RnBeads were analyzed using JADBio in order to construct an accurate model specific for tracing BrCa. The original dataset (218 BrCa tissues and 193 healthy tissues) was automatically and randomly split into a training dataset of 151 BrCa and 131 healthy tissues and a validation dataset of 66 BrCa and 55 healthy tissues. Analysis of the training dataset of 29,703 gene array features produced one signature containing 5 features via a support vector machines (SVM) algorithm (https://app.jadbio.com/share/4fd50c38-d0a1-4f28-96c9-480b29b4a3e2, accessed on 1 October 2021). Three of them were protein-coding genes, namely, *CCDC181*, *HIST2H3PS2* and *CFTR*, and two were RNA genes, namely, *RUVBL1-AS1* and *AL161908.1* (Table 1). All genes presented increased methylation in BrCa in relation to healthy tissues/cells. In discriminating BrCa against healthy tissues, this signature reached an area under the curve (AUC) of 0.987 (0.963–1.000) and an average precision of 0.987 (0.955–1.000) (Figure 2A). Upon validation in the test dataset, the model showed an AUC and an average precision of 0.995 (Figure 2A), verifying the model’s performance metrics. The performance and inspection results are depicted in Figure 2B–D.

#### 2.1.4. Validation and Applicability of BrCa-Specific Methylation Biosignature on ccfDNA

To validate the discrimination performance of the BrCa-specific five-feature biosignature on ccfDNA and its applicability to liquid biopsy, we applied it to an external independent dataset of three BrCa ccfDNA samples and five ccfDNA samples from age-matched healthy women. The analysis revealed the model’s AUC and an average precision of 1.000 (Figure 2E,F).

#### 2.1.5. Biological Relevance of Genes Selected in the BrCa-Specific Methylation Biosignature

Feature selection performed via ML identifies a minimum subset of features bearing the maximal classifying ability between groups. In tasks such as the one addressed here, i.e., to build a tissue-specific methylation biosignature, it is interesting to know if the DMGs included in the model have an established role in the related pathophysiology as revealed by their biological characteristics. All five DMGs of the BrCa biosignature were subjected to GO analysis using the GeneCards database (Table 1). *CCDC181*’s molecular function is related to microtubule binding, while it is mainly found in the manchette and cytoplasm. *HIST2H3PS2*’s molecular function is associated with DNA binding and protein heterodimerization activity and is mainly found in nucleus and on chromosome. CFTR’s molecular function is related, among other things, to nucleotide binding and chloride channel activity, and it is located in the nucleus, cytoplasm and in other cellular components and participates in cholesterol biosynthesis, ion and chloride transport among other things. For *RUVBL1-AS1* and *AL161908.1*, no information was found in the GeneCards database (Table 1).

Furthermore, in order to examine if the protein products of the three protein-encoding DMGs included in the BrCa-specific biosignature were somehow implicated in BrCa pathophysiology, we analyzed the identified genes, using a literature mining tool UniReD, which predicts functional associations between proteins. As previously [17], for this analysis, we used the following list of 10 protein-coding genes with an established role in BrCa pathophysiology, namely, *BRCA1* [26], *BRCA2* [26], *RASSF1* [27], *ESR1* [28], *TP53* [29], *PIK3CA* [30], *BRMS1* [31], *CDH1* [32], *CST6* [33] and *PTEN* [34]. All genes were found to be associated with breast cancer pathways according to the KEGG pathway identification. *CFTR* reached a score of 7, while *CCDC181* reached a score of 5 and *HIST2H3PS2* a score of 1, showing fewer known associations (Table 1).

### 2.2. Osteoarhtitis

#### 2.2.1. Differential Methylation Analysis Comparing OA and Healthy Tissues

**Figure 3:**
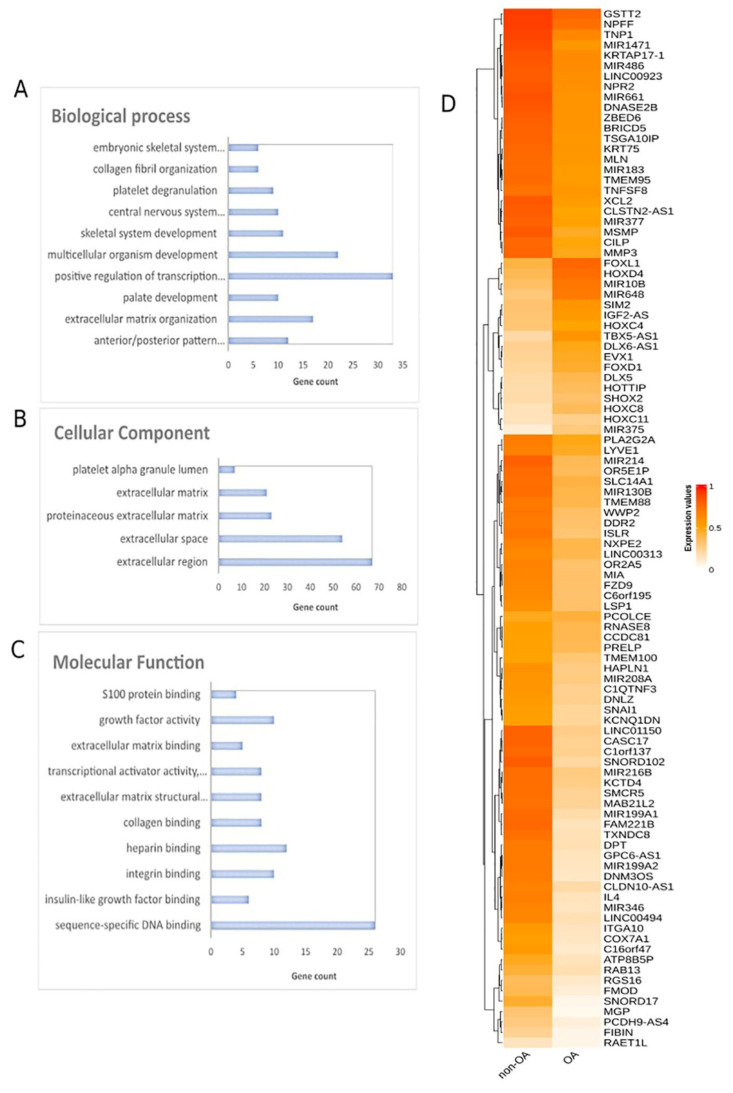
Differential methylation analysis comparing OA and healthy tissues. Gene ontology analysis of top 400 DMGs in the aspects of (**A**) biological process, (**B**) cellular component and (**C**) molecular function analysis. (**D**) Heatmap plot of top 100 DMGs comparing OA and healthy tissues. Abbreviations: OA = osteoarthritis, DMGs = differentially methylated genes.

Methylomes of OA cartilage tissues were analyzed in comparison to healthy tissues, including healthy cartilages, breast, blood, liver, muscle, colon, gastric, lung and adipose (Supplementary Table S1). Raw data from 151 OA cartilages tissues and 216 healthy tissues were subjected to RnBeads for differential methylation analysis and 18,413 DMGs (FDR < 0.05) emerged. Among those, 12,400 DMGs were found to be hypomethylated, while 6013 were found to be hypermethylated in OA in relation to healthy tissues. A heatmap of DMGs is presented in Figure 3. Further, the 400 top-ranking DMGs based on FDR were chosen for functional analysis. Of these, 354 were hypomethylated, and the remaining 56 were hypermethylated in OA in relation to healthy tissues. The complete list of the 400 top-ranking DMGs is presented in Supplementary Table S3.

#### 2.2.2. Functional Analysis of DMGs Comparing OA and Healthy Tissues

Gene ontology analysis of the 400 DMGs was conducted using the DAVID tool (Figure 3A–C). Molecular function analysis showed enrichment in sequence-specific DNA binding, insulin-like growth factor binding, integrin binding, heparin binding and collagen binding. Regarding biological process enrichment analysis, DMGs were found to participate mainly in anterior/posterior pattern specification and in extracellular matrix organization. Cellular component analysis of the studied genes showed extracellular region, extracellular space, proteinaceous extracellular matrix and extracellular matrix enrichment. Further, Reactome analysis via ConsensusPathDB mainly revealed enrichment in metabolism, extracellular matrix organization and signal transduction (Supplementary Figure S2). The PPI network of the 400 DMGs is presented in Supplementary Materials.

#### 2.2.3. OA Specific Methylation Biosignature through AutoML

**Figure 4:**
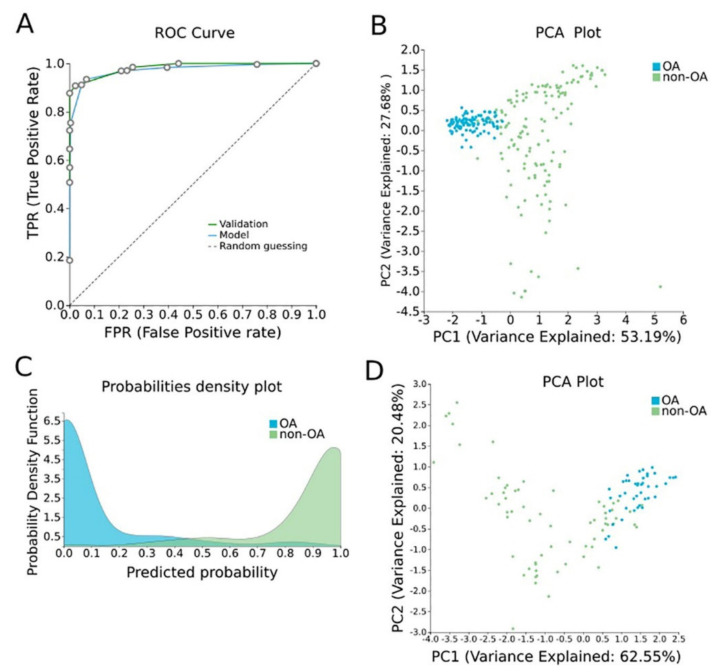
OA-specific methylation biosignature built using AutoML. (**A**) ROC curves of training (blue line) and validation (green line) models. (**B**) Supervised PCA plot (i.e., only considering the selected relevant biomarkers) presents separation between OA (blue) and non-OA healthy tissues (green) within the training group. (**C**) Out-of-sample probability density plot (i.e., probability predictions when samples were not used for training) depicts discrete distributions among studied classes of the training group. (**D**) PCA plot presents separation between OA (blue) and non-OA healthy tissues (green) within the validation group. Abbreviations: OA = osteoarthritis, ROC = receiver operating characteristic, PCA = principal component analysis.

In order to construct a specific model for OA, β-values were uploaded to JADBio. The original dataset (151 OA tissues and 216 healthy tissues) was automatically and randomly split into a training dataset of 108 OA and 144 healthy tissues and a validation dataset of 43 OA and 65 healthy tissues. An analysis of the training dataset of 29,585 gene array features produced three equivalent signatures containing 4 features each via a classification random forests algorithm (https://app.jadbio.com/share/2fee0023-8330-4b54-ab0c-ddbaf032b506, accessed on 1 October 2021). Two of them were protein-coding genes, namely *CASD1* and *STOML1*, two were lncRNA genes, namely, *LINC01350* and *RP11-272L13.3*, and one was an RNA gene, namely, *CARMAL*. The last was the *RP11-515E23.2* gene (Table 2). Common features between models were *RP11-515E23.2*, *LINC01350* and *CASD1*. All genes showed the down-regulation of methylation in OA cartilage in relation to healthy tissues. In discriminating OA against healthy tissues, signatures reached an AUC of 0.978 (0.942–1.000) and average precision of 0.986 (0.962–1.000) (Figure 4A). Upon validation, the model showed an AUC of 0.990–0.995 and an average precision of 0.994–0.997 (Figure 4A), verifying the stability and accuracy of its estimation. Performance validation and inspection are depicted in Figure 4B,C.

#### 2.2.4. Biological Relevance of Genes Selected in the OA-Specific Methylation Biosignature

GO analysis revealed the biological characteristics of the genes included in the assembled models. *CASD1* participates in acetyltransferase and transferase activity molecular functions and others, is mainly located in the Golgi system and is involved in the carbohydrate metabolic process. *STOML1* takes part in protein binding, is mainly located in the endosome and plasma membrane and participates in lipid transport (Table 2). For *LINC01350*, *RP11-515E23.2*, *CARMAL* and *RP11-272L13.31*, no relevant information was found in the GeneCards database (Table 2).

Following this, the two protein-coding gene features were analyzed via UniReD using a list of 10 protein-coding genes that are known to be related to OA pathophysiology, namely, *VDR* [35], *AGC1* [36], *IGF-1* [37], *ADAMTS4* [38], *TGF beta* [39], *MATN3* [40], *MMP13* [41], *COL2A1* [42], *COL11A1* [43] and *COL9A1* [44]. Only *STOML1* was found to be associated with OA pathways according to the KEGG pathway identification, reaching a score of 2.5 (Table 2).

### 2.3. Diabetes

#### 2.3.1. Differential Methylation Analysis Comparing Pancreatic β-Cells and Other Tissues

**Figure 5:**
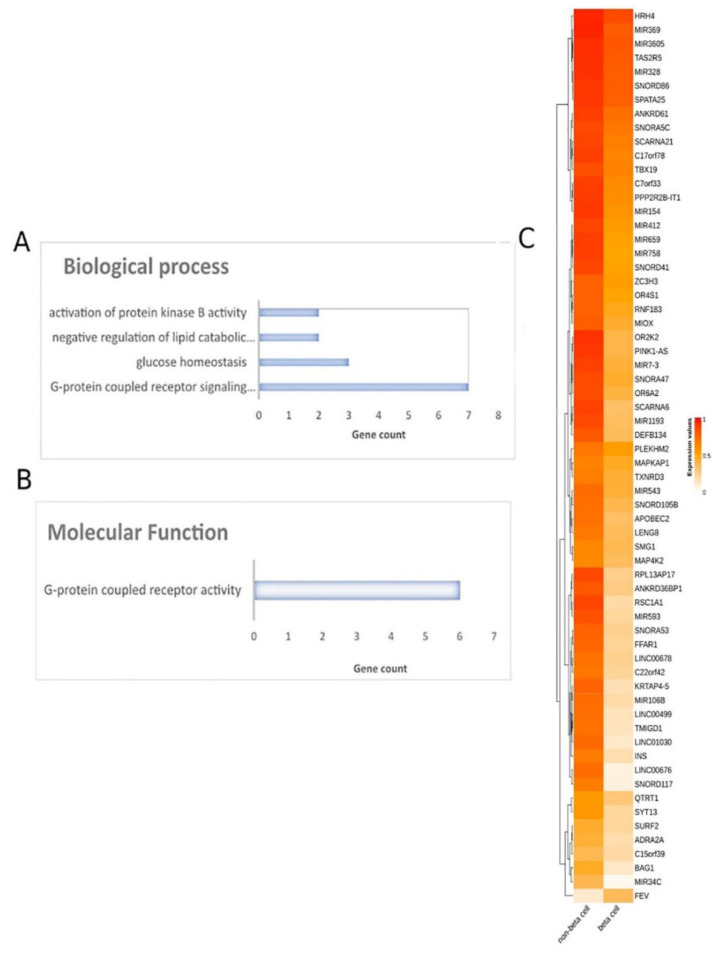
Differential methylation analysis comparing pancreatic β-cells and other tissues. Gene ontology analysis of 66 DMGs in the aspects of (**A**) biological process and (**B**) molecular function analysis. (**C**) Heatmap plot of 66 DMGs comparing pancreatic β-cells and other healthy tissues. Abbreviations: DMGs = differentially methylated genes.

To decipher the methylation landscape of pancreatic β-cells, which could be of value in monitoring diabetes, raw methylomes of 3 pancreatic β-cell samples were analyzed against 28 other tissues/cell types, including blood, serum, muscle, adipose, spleen, colon, gastric, liver, skin, etc. (Supplementary Table S1) using RnBeads. Differential methylation analysis revealed 65 hypomethylated and 1 hypermethylated genes in β-cells in comparison to other tissues (FDR < 0.05). A heatmap of the emergent DMGs is presented in Figure 5. The complete list of the 66 DMGs is presented in Supplementary Table S4.

#### 2.3.2. Functional Analysis of DMGs Comparing Pancreatic β-Cells and Other Tissues

Further, all DMGs identified were subjected to functional analysis. Molecular function analysis showed an enrichment in the G-protein-coupled receptor activity and signaling pathway, glucose homeostasis, the negative regulation of lipid catabolic process and the activation of protein kinase B activity (Figure 5). Reactome pathway analysis did not lead to any pathways. The PPI network of the 66 DMGs is presented in Supplementary Materials.

#### 2.3.3. Pancreatic β-Cell Specific Methylation Biosignature Using AutoML

**Figure 6:**
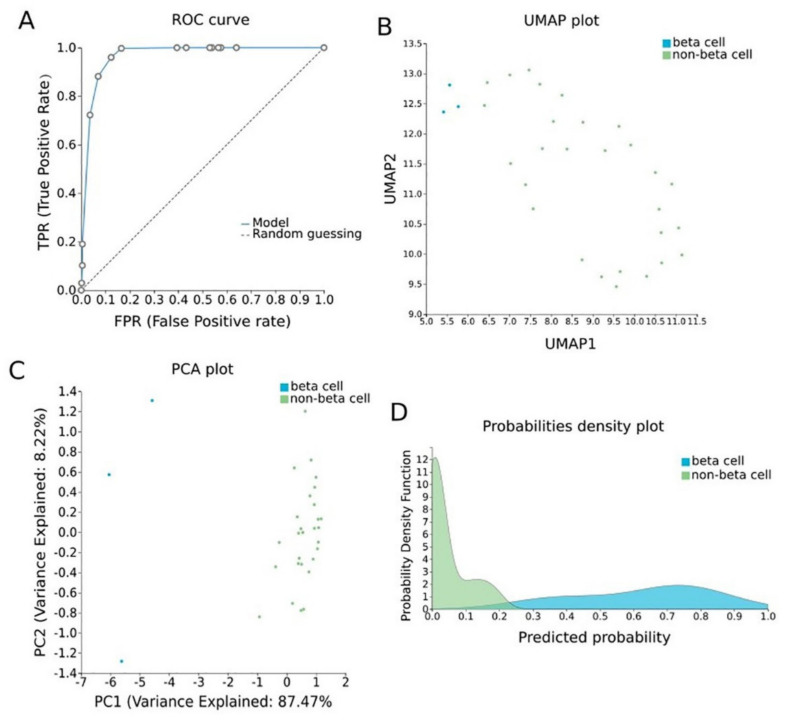
Pancreatic β-cell-specific methylation biosignature built using AutoML. (**A**) ROC curve of model. (**B**) UMAP plot shows separation between pancreatic β-cells (blue) and other tissues (green). (**C**) Supervised PCA plot (i.e., only considering the selected relevant biomarkers) presents separation between pancreatic β-cells (blue) and other tissues (green). (**D**) Out-of-sample probability density plot (i.e., probability predictions when samples were not used for training) depicts discrete distributions among studied classes. Abbreviations: ROC = receiver operating characteristic, PCA = principal component analysis, UMAP = uniform manifold approximation and projection.

To construct a pancreatic β-cell-specific methylation biosignature, methylome β-values of 3 β-cell samples and 28 other tissue/cell samples were analyzed through JADBio. From the 28,021 CG feature dataset, AutoML analysis produce a biosignature containing 4 features via a support vector machine algorithm (https://app.jadbio.com/share/7ebbc7c3-b861-41af-8a39-88202756d609, accesed on 1 October 2021). Two of them were protein-coding genes, namely, *TXNRD3* and *LENG8*, one was a snoRNA gene, namely, *SCARNA6*, and one an LncRNA gene, namely, AC008741.1 (Table 3). All genes showed decreased methylation in pancreatic β-cells in relation to other tissues/cells. The signature’s performance in discriminating β-cells reached an AUC of 0.984 (0.909–1.000) and an average precision of 0.995 (0.975–1.000) (Figure 6A). The model’s performance and inspection are depicted in Figure 6B,C.

#### 2.3.4. Biological Relevance of Genes Selected in the β-Cell-Specific Methylation Biosignature

GO analysis revealed that *SCARNA6* is a nucleolus component and is involved in RNA processing. *TXNRD3* has a nucleotide binding function, thioredoxin disulfide reductase activity, electron transfer activity and others (Table 3). It is a component of nucleoplasm and cytoplasm and is involved in many biological processes, such as cell differentiation. *LENG8* participates in protein binding in the nucleus. For AC008741.1 no information about its molecular function, cellular component and biological process was available in the GeneCards database (Table 3).

Finally, the two protein-coding gene features were analyzed with UniReD, using a list of 10 protein-coding genes that are known to be related to diabetes pathophysiology—*SLC2A2* [45], *IAPP* [46], *GSK* [47], *INSR* [48], *IRS1* [49], *PPARG* [50], *KCNJ11* [51], *ABCC8* [52], *TCF7L2* [53] and *FTO* [54]. Only *TXNRD3* was found to be associated with diabetes-related pathways according to the KEGG pathway identification, reaching a score of 5.5 (Table 3).

## 3. Discussion

A major burden on the implementation of liquid biopsy diagnostics in cancer and other pathologies is the lack of a means to identify a tissue-specific fraction of the bulk of ccfDNA in biological fluids. In this study, we hypothesize that this problem can be effectively addressed by studying gene methylation, which is, in principle, a tissue-specific event. We compared methylomes of the major tissue or cell types involved in a pathology against methylomes from multiple healthy tissues of the body which may contribute to the ccfDNA pool in the circulation in an effort to determine its heterogenicity. With multiple bioinformatic analyses, we aimed to identify those methylation features which are specific to the tissue and should be mirrored in the ccfDNA released there. We used three distinct pathological conditions as use cases, i.e., one malignancy (breast cancer, BrCa), one metabolic (diabetes) and one inflammatory (osteoarthritis, OA).

In the case of BrCa, the comparison between BrCa tissues vs. healthy tissues resulted in 19,248 DMGs, the majority of them being hypermethylated in cancer. Functional analysis showed that the most dysregulated genes have G-protein-coupled receptor (GPCRs) and transcriptional activator activity. Indeed, it has been shown that GPCRs are involved in the development and progression of many tumours, including breast cancer [55]. Additionally, the activation of transcription is a critical event in BrCa pathophysiology [56,57]. DMGs were found to be highly involved in sensory perception pathways, previously connected to cancer and the side effects of cancer treatment [58].

Using AutoML, we were able to construct a five-gene signature exhibiting a high AUC of 0.987 and a precision of 0.987 when discriminating BrCa against healthy tissues. Three of them were protein-coding genes, namely, *CCDC181*, *HIST2H3PS2* and *CFTR,* and two were novel RNA genes. According to UniRed analysis, more associations to known BrCa pathways were found for *CFTR* and less for *CCDC181* and *HIST2H3PS2.* Indeed, previous studies have shown that the aberrant methylation of *CTFR* has been correlated to the prognosis and diagnosis of BrCa [59], as well as to bladder cancer [60], hepatocellular carcinoma [61] and lung cancer [62]. Furthermore, *CCDC181* methylation has been suggested to be a prognostic biomarker in prostate cancer [63] and lung cancer [64]. Especially in BrCa, *CCDC181* methylation was suggested as a biomarker with which to estimate the breast cancer cell fraction in tissue samples [65], corroborating our results. The methylation of *HIST2H3PS2* has been linked to endometrial cancer tissue [66], but no association to BrCa has been previously reported. Based on our results, its involvement in breast malignancy is worthy of further attention.

In order to examine if ccfDNA reflects the specific methylation pattern of BrCa tissues, we validated our five-gene biosignature in an independent, external ccfDNA BrCa dataset. The model showed an AUC and precision of 1.000 in discriminating ccfDNA of BrCa patients from that of healthy women, confirming the hypothesis that ccfDNA mirrors reliably the specific methylation profile of the tissue of origin. This hypothesis has also been confirmed in previously studies produced either by computational approaches or experimental [25,67,68].

Our results also verify the translational value of the BrCa-specific five-gene methylation biosignature in clinical practice as a tool for diagnosing/monitoring tumor burden in liquid biopsies. In fact, its in silico-demonstrated classifying performance in terms of specificity/sensitivity is higher than others previously reported [69,70,71]. For example, we have previously reported a ccfDNA biosignature including five gene methylation features and ccfDNA levels with an AUC of 0.844 [71]. Further validation in a real-world clinical setting will confirm the credibility of our data-driven approach in building classifiers readily available to be applied in diagnostics.

In the case of OA, when OA cartilage tissues were compared to healthy tissues, 18,413 DMGs emerged, the majority of them being hypomethylated in OA. Functional analysis showed that DMGs were enriched in insulin-like growth factor binding, integrin binding and collagen binding functions. In accordance to our findings, it is known that insulin-like growth factors are implicated in OA and have a prognostic value [72]. Additionally, integrin dysfunction [73] and collagen degradation [74] are well-known pathways involved in OA pathogenesis. In the biological process analysis, the identified DMGs were found to participate mainly in anterior/posterior pattern specification and in extracellular matrix (ECM) organization. Indeed, increased catabolism in the extracellular matrix of the articular cartilage is a key factor in the pathogenesis of OA [75].

Most importantly, AutoML analysis delivered three equivalent OA cartilage-specific biosignatures with high performance (AUC of 0.978 and precision of 0.986) containing four features each. Two of them were protein-coding genes, namely, *CASD1* and *STOML1*, two were lncRNA genes, namely, *LINC01350* and *RP11-272L13.3*, one was an RNA gene, namely, *CARMAL*. Between them, only *STOML1* was found to be associated with known OA pathways, reaching a score of 2.5 through text mining. In fact, stomatin-like (STOML) protein family members are found to be overexpressed in OA [76]. No associations were found between OA and the other signature’s genes, *CASD1*, the two lncRNA genes and the *CARMAL* RNA gene, either using a machine learning-aided or manual search of the literature. Thus, the expression and biological relevance of these genes in OA pathophysiology, as well as their potential as novel biomarkers, should be investigated, as their methylation was highlighted to hold great classifying capacity in the OA biosignatures.

Specific methylation patterns of pancreatic β-cells would be of great value in the early detection and monitoring of pancreatic cell loss during diabetes. Differential methylation analysis comparing pancreatic β-cells and other tissues revealed 66 DMGs, 65 of them being hypomethylated in β-cells. Interestingly, through molecular function analysis, DMGs were associated with G-protein-coupled receptor (GPCR) activity and signaling pathway and protein kinase B (PKB) activity. Many GPCRs are involved in the development of insulin resistance and pancreatic β-cell dysfunction, which can lead to obesity-induced T2DM [77]. Additionally, it has been shown that the β-cell expression of PKB in mice increases β-cell mass by preserving β-cell survival [78]. Not surprisingly, DMGs were also found to be associated with glucose homeostasis, as blood glucose levels are tightly controlled by the regulation of insulin release from pancreatic β-cells [79].

Next, a highly performing biosignature (AUC of 0.984 and precision of 0.995) was developed through AutoML analysis. The biosignature contained two protein-coding genes, namely, *TXNRD3* and *LENG8*, one snoRNA gene and one LncRNA gene. Only *TXNRD3* was found to be associated with diabetes-related pathways, reaching a score of 5.5 in machine learning-aided text mining. Indeed, in a recent study of animal models, a combination of hyperglycemia, long-term insulin resistance and obesity was linked to reduced mRNA expression of thioredoxin reductase 3 (Txnrd3) along with selenoprotein Gpx3 and selenophosphate synthetase 2 (Sephs2) in adipose tissue [80]. In addition to that, it has been found that thioredoxin reductase is the primary mediator of thioredoxin reduction in β-cells [81].

Several studies have shown that liquid biopsy biomaterials such as ccfDNA retain the tissue/cell- or disease-specific methylation profile, opening the way for biomarker discovery [17,71]. Gene methylation panels examined in liquid biopsy are implemented for the clinical management of some diseases, limited so far to a few cancer types [82,83,84]. To this end, building new highly performing panels that can be applicable to ccfDNA is of utmost importance. Unfortunately, in the cases of OA and diabetes, ccfDNA methylome datasets were not available to allow the in silico validation of our biosignatures in liquid biopsies, as in the case of BrCa. It would also be very interesting to analyze diabetic pancreatic β-cell methylomes, should they become available.

A few previous studies have also tried to identify unique methylation patterns specific to different pathological tissues. Moss et al. compared the genome-wide methylation profiles of normal breast and breast cancer tissue to those of other normal and cancerous tissues and identified CpG sites with breast-unique methylation patterns. A three-marker biosignature was suggested for BrCa diagnosis [85]. In addition, Zemmour et al. compared the methylomes of human heart chambers to the methylomes of 23 other human tissues in order to identify cardiomyocyte-specific biomarkers for the diagnosis of acute myocardial infarction [86]. Additionally, Lehmann-Werman et al. compared multiple human tissue methylomes and selected three hepatocyte-specific methylation markers, which were unmethylated in the liver as compared to other tissues and cell types for monitoring liver damage [87]. Here, in order to build methylation-based biosignatures, we employed, for the first time, AutoML using JADBio. As we have previously shown [17,18,88], this approach presents two advantages of major significance for further developments in biomarker discovery: (1) It has high-performing classifiers with low feature numbers via feature selection, i.e., automatic calculations for identifying the minimum feature number within a dataset of some thousands of features that retain the maximum classifying power. Reducing the dimensions of a signature is a great advantage in terms of translatability to cost-effective assays with less technical requirements for multiplexing, moving from the multi-dimensional omics results to simpler classifiers. Upon prospective clinical validation, these signatures can offer feasible solutions for laboratory tests that could be realized in any standardly equipped diagnostic lab. (2) JADBio has been shown to shield against typical methodological pitfalls in data analysis that lead to overfitting and overestimating performance and, therefore, to misleading results. This is again confirmed here, as the AUC of the biosignatures built did not fall significantly when tested in the validation sub-datasets or in independent cohorts, adding credibility to this approach, showing that it can deliver mature solutions for clinical development.

## 4. Materials and Methods

### 4.1. Data Sources

**Figure 7:**
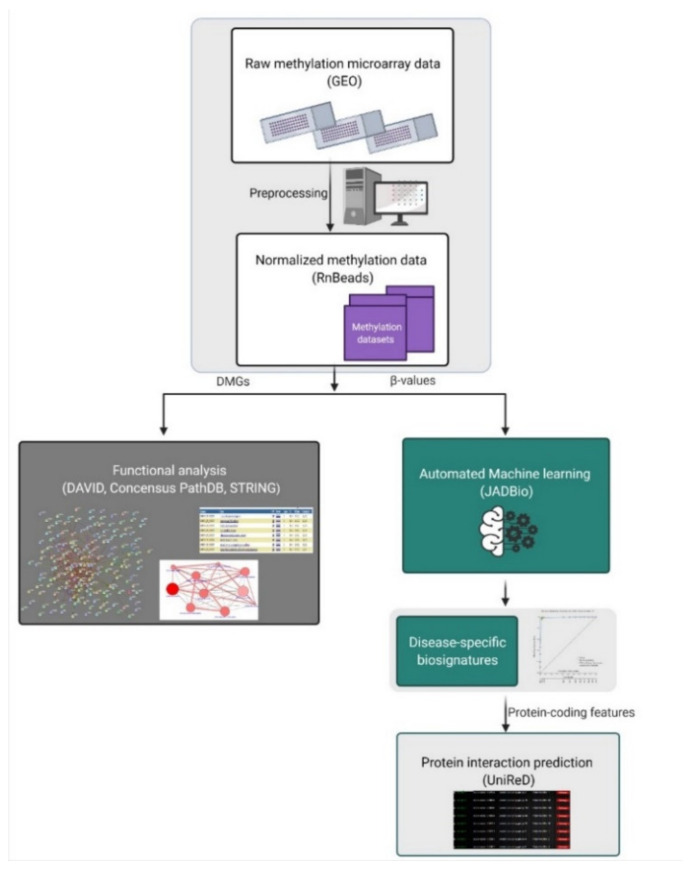
Study workflow. Abbreviations: DMGs = differentially methylated genes, GEO = Gene Expression Omnibus. Created with BioRender.com, accessed on 20 July 2021.

Raw DNA methylation data from several tissues and cell types and corresponding demographic and clinical data were retrieved from the GEO database [89]. GEO sample inclusion criteria were: (1) platform: Infinium Human Methylation 450K bead-chip array, (2) available raw data. The GEO database was searched using several human tissues and cell types, such as blood, spleen, brain, breast, pancreas, adipose, pancreatic beta cells, alpha cells, T cells, etc., as keywords and ‘Methylation profiling by array’ as the study type. In total, 45 studies were chosen, and 430 tissue samples were downloaded. GEO studies and corresponding tissues/cell types used in our study are presented in Supplementary Table S1. An analysis was performed for each of the 3 studied pathologies separately: (1) BrCa tissues vs. healthy tissues/cell types, (2) OA cartilage tissues vs. healthy tissues/cell types, (3) pancreatic β-cells vs. other healthy tissues/cell types. The study workflow is depicted in Figure 7.

### 4.2. Data Preprocessing and Differential Methylation Analysis

Raw DNA methylation data (IDAT files) and sample annotation files were subjected to the Bioconductor R package RnBeads v2.0 [90] and processed as performed previously by our team [17]. In our workflow, genes were chosen as the genomic region of interest. Methylation β values are expressed as decimal values between 0.0 (no methylation) and 1.0 (full methylation). Differentially methylated genes (DMGs) were ranked based on the false discovery rate (FDR) adjusted *p*-value for further downstream analysis. The first 100 DMGs in osteoarthritis and breast cancer and all the DMG in the case of β-cells were plotted in heatmaps using the R programming environment (R version 3.6.1) with the ComplexHeatmap package. Methylation values of DMGs were clustered using the default hierarchical clustering (hclust) method.

### 4.3. Automated Machine Learning Analysis (AutoML)

The AutoML technology Just Add Data Bio (JADBio) [22] was used to produce disease/tissue-specific biosignatures based on the β-value methylation data. JADBio is applicable to low-sample, high-dimensional omics data and provides predictive models by employing standard, best-practice, and state-of-the-art statistical and machine learning methods. JADBio automatically produces predictive models either for a discrete (classification), or a continuous (regression) or a time-to-event (survival analysis) outcome. Specifically, JADBio has the following functionality and properties: (a) Given a 2D matrix of data, it automatically produces predictive models for a categorical (classification), continuous (regression) or time-to-event (survival analysis) outcome. No selection of appropriate algorithms to apply is necessary, nor is a tuning of their hyper-parameter values; it is performed automatically. Available classification algorithms are: classification random forests, support vector machines (SVM), ridge logistic regression and classification decision trees. (b) It identifies multiple equivalent biosignatures, i.e., subsets of selected biomarkers. The algorithms used for biosignature identification (i.e., feature selection) are currently SES [91] and Lasso [92]. (c) It produces conservative predictive performance estimates and corresponding confidence intervals by employing out-of-sample estimation protocols, such as variants of K-fold cross-validation. It reliably processes up to hundreds of thousands of features and sample sizes as low as a couple of dozen. JADBio also employs the recently developed BBC-CV protocol for tuning the hyper-parameters of algorithms while estimating the performance and adjusting for multiple tries. For all analyses, the performance was estimated via internal validation after correcting for the “winner’s curse” and the fact that multiple machine learning pipelines are tried using the BBC-CV algorithm [93]. JADBio has been evaluated many times on hundreds of omics datasets with respect to predictive performance, number of biomarkers selected and correctness of predictive performance estimation [22]. In the same paper, it is compared against the previous state-of-the-art AutoML tools.

In our analysis, extensive tuning effort was used as a preference when running the tool, and large sample datasets were automatically split into training and validation groups in a proportion of 70/30 by JADBio. The maximum size of biosignatures was set to be up to 5 features for better applicability in clinical practice.

Gene description and the biological characteristics of each gene feature based on gene ontology (GO) analysis were retrieved by the GeneCards database [94].

### 4.4. Biological Association Analysis through Text Mining

We employed UniProt Related Documents (UniReD) to analyze the protein-coding features of the assembled biosignatures. UniReD is a computational tool used to predict functional relationships between proteins based on a machine learning algorithm called mcl [95]. The relationships are extracted using biomedical literature. UniReD includes information only for reviewed UniProt proteins and for organisms *Homo sapiens* and *Mus musculus*. UniReD computes a score for each protein under investigation signifying the relatedness to a specific pathway.

Using UniRed, we tested the associations of identified features against ten genes known for their significant implication in BrCa, OA, and pancreatic β-cell function/diabetes pathways (UniReD uses KEGG pathway analysis system). We ran a UniReD analysis for each protein-coding feature and searched the list of 10 protein-coding genes related to each pathology to see whether we could find an association. When we could not find the human protein, we searched for homologs of the protein or we ran UniReD using the mouse ortholog and we conducted the same analysis. We used a simple scoring system, i.e., we assigned 1 point when we found the human protein. If we could not find an exact match, we assigned 0.5 points whenever we were able to find a homolog of the protein in a human. If we were still not able to find a protein of the same family in a human, we conducted the analysis using the mouse ortholog and we assigned 0.5 points when we were able to find one. The literature searching of biosignature genes was performed using BioTextQuest(+), a platform for knowledge integration, literature mining and concept discovery [96].

### 4.5. Functional Analysis of DMGs

The biological functions of the 400 top-ranked DMGs were further investigated using publicly available tools. The Database for Annotation, Visualization and Integrated Discovery (DAVID) v6.8 [97] was used for gene ontology (GO) analysis of DMGs according to the following categories: biological process, cellular component and molecular function. Benjamini–Hochberg FDR < 0.05 was set as the cutoff criterion in GO analysis. In case of the β-cells/diabetes analysis, due to the small number of comparisons, a *p*-value < 0.01 was set as a cutoff level of significance. In addition, we used ConsensusPathDB-Human Release 34 [98] to perform Reactome analysis. Finally, in order to evaluate the relationships among DMGs, we analyzed them using the Search Tool for the Retrieval of Interacting Genes (STRING) v11.0 [99] and protein–protein interaction (PPI) networks were visualized using Cytoscape 3.8.2 [100].

### 4.6. Evaluation of Biosignatures on Liquid Biopsy

In order to examine the performance of the assembled biosignatures on liquid biopsy biomaterial, we searched the GEO database for related datasets. ‘Liquid biopsy’, ‘cell free DNA’ ‘ccfDNA’, ‘circulating DNA’ and ‘ctDNA’ were used as keywords in the GEO query and ‘Methylation profiling by array’ as the study type. In total, 4 studies were found. However, only one study, GSE122126 [23], contained suitable and adequate ccfDNA samples against which to test the BrCa biosignature.

### 4.7. Statistical Analysis

The Kolmogorov–Smirnov test was applied in order to check the normality of age distribution among groups. A *t*-test was then used to compare the mean age among groups. Statistical significance was set at *p*-value < 0.05. Statistical analysis was performed using the IBM SPSS Statistics 21 software (IBM Corp. 2010. IBM SPSS Statistics for Windows, Version 21.0. Armonk, NY, USA).

## 5. Conclusions

Revisiting available microarray methylomes and using an innovative AutoML tool, we were able to produce three simple biosignatures for clinical implementation in the management of BrCa, OA and diabetes. They showed high performance in discriminating the tissues of interest among the bulk of tissues of different origin. The data-driven approach presented here can be extrapolated to any other pathological condition, given that the major tissue or cell type involved in its pathogenesis is known and contributes significantly in the ccfDNA pool of circulation, and there are available methylomes. Most importantly, the validation of the BrCa-specific biosignature in an independent ccfDNA dataset confirmed the potential for application in liquid biopsy diagnostics. Our immediate plans are to test the applicability of the constructed models in ccfDNA samples through multiplex PCR (Methylight, ddPCR) assays and/or targeted next-generation sequencing for further clinical development. Furthermore, our in-depth analysis of the methylomes via functional analysis of the identified DMGs, and in particular the biological relevance of those selected in the biosignatures via text mining, unraveled novel insights into the pathophysiological pathways of the studied conditions and augmented knowledge exploitation.

## Figures and Tables

**Table 1:** Differentially methylated genes selected in the BrCa-specific signature built using AutoML analysis. Their biological characteristics and functions revealed by GO analysis as well as their methylation status are described.

Signature Genes	Gene Type	Description	Pathway	GO—Molecular Function	GO—Cellular Components	GO—Biological Process	UniReD Score	Methylation in BrCa in Relation to Healthy Tissues
*CCDC181*	Protein Coding	Coiled-Coil Domain Containing 181	NA	microtubule binding	manchette, cytoplasm, cytoskeleton, microtubule, cilium	NA	5	Hypermethylation
*HIST2H3PS2*	Protein Coding	Histone Cluster 2, H3, Pseudogene 2	NA	DNA binding, protein heterodimerization activity	Nucleus, Chromosome	NA	1	Hypermethylation
*RUVBL1-AS1*	RNA Gene	RUVBL1 Antisense RNA 1	NA	NA	NA	NA	NA	Hypermethylation
*CFTR*	Protein Coding	CF Transmembrane Conductance Regulator	CDK-mediated phosphorylation and removal of Cdc6, bacterial infections in CF airways, regulation of CFTR activity, salivary secretion	nucleotide binding, chloride channel activity, intracellularly ATP-gated chloride channel activity	nucleus, cytoplasm, lysosomal membrane, endsome, early endsome	cholesterol biosynthetic process, ion transport, chloride transport, vesicle docking involved in exocytes	7	Hypermethylation
*AL161908.1*	RNA Gene	Novel Transcript, Antisense To LIM1B	NA	NA	NA	NA	NA	Hypermethylation

Abbreviations: BrCa: breast cancer, AutoML: automated machine Learning, GO: gene Ontology, NA: non-available.

**Table 2:** Differentially methylated genes selected in the OA cartilage-specific signature built using AutoML analysis. Their biological characteristics and functions revealed by GO analysis as well as their methylation status are described.

Signature Genes	Gene Type	Description	Pathway	GO—Molecular Function	GO—Cellular Components	GO—Biological Process	UniReD Score	Methylation in OA in Relation to Other Tissues
*CASD1*	Protein Coding	CAS1 Domain Containing 1	NA	acetyltransferase activity, transferase activity, transferring acyl groups	Golgi membrane, Golgi apparatus, membrane, integral component of membrane, integral component of Golgi membrane	Carbohydrate metabolic process	0	Hypomethylation
*LINC01350*	LncRNA	Long Intergenic Non-Protein Coding RNA 1350	NA	NA	NA	NA	NA	Hypomethylation
*RP11-515E23.2*	NA	NA	NA	NA	NA	NA	NA	Hypomethylation
*STOML1*	Protein Coding	Stomatin-Like 1	NA	protein binding	endosome, plasma membrane, membrane, integral component of membrane	lipid transport	2.5	Hypomethylation
*CARMAL*	RNA Gene	Coronary Artery Disease Region-Linked MFGE8 Regulatory LncRNA	NA	NA	NA	NA	NA	Hypomethylation
*RP11-272L13.3*	LncRNA	NA	NA	NA	NA	NA	NA	Hypomethylation

Abbreviations: OA: osteoarthritis, AutoML: automated machine learning, GO: gene ontology, NA: non-available.

**Table 3:** Differentially methylated genes selected in the pancreatic β-cell-specific signature built using AutoML analysis comparing methylomes of β-cells and other healthy tissues. Their biological characteristics and functions revealed by GO analysis as well as their methylation status are described.

Signature Genes	Gene Type	Description	Pathway	GO—Molecular Function	GO—Cellular Components	GO—Biological Process	UniReD Score	Methylation in Pancreatic β Cells in Relation to Other Healthy Tissues
*SCARNA6*	snoRNA	Small Cajal Body-Specific RNA 6	NA	NA	nucleolus	RNA processing	ΝA	Hypomethylation
*TXNRD3*	Protein Coding	Thioredoxin Reductase 3	folate metabolism and mechanisms of CFTR activation by S-nitrosoglutathione	nucleotide binding, thioredoxin disulfide reductase activity, electron transfer activity, protein disulfide oxidoreductase activity	cell, nucleoplasm, cytoplasm, endoplasmic reticulum, cytosol	multicellular organism development, spermatogenesis, electron transport chain, cell differentiation	5.5	Hypomethylation
*AC008741.1*	lncRNA	Novel Transcript, Antisense To ZKSCAN2	NA	NA	NA	NA	ΝA	Hypomethylation
*LENG8*	Protein Coding	Leukocyte Receptor Cluster Member	NA	protein binding	nucleus	NA	NA	Hypomethylation

Abbreviations: AutoML: automated machine learning, GO: gene ontology, NA: non-available.

## Data Availability

All data analyzed in this study are publicly available.

## Supplementary Materials

The following supporting information can be downloaded at: https://www.mdpi.com/article/10.3390/ijms23062959/s1.
