# Supplementary material for: Tissue-Specific Methylation Biosignatures for Monitoring Diseases: An In Silico Approach
Source: Int J Mol Sci. 2022 Mar 9;23(6):2959. doi: 10.3390/ijms23062959 (PMC8952417; doi:10.3390/ijms23062959)
Supplement: Supplementary file 1 [file ijms-23-02959-s001.zip › ijms-1612001-supplementary.pdf]

Supplementary Information

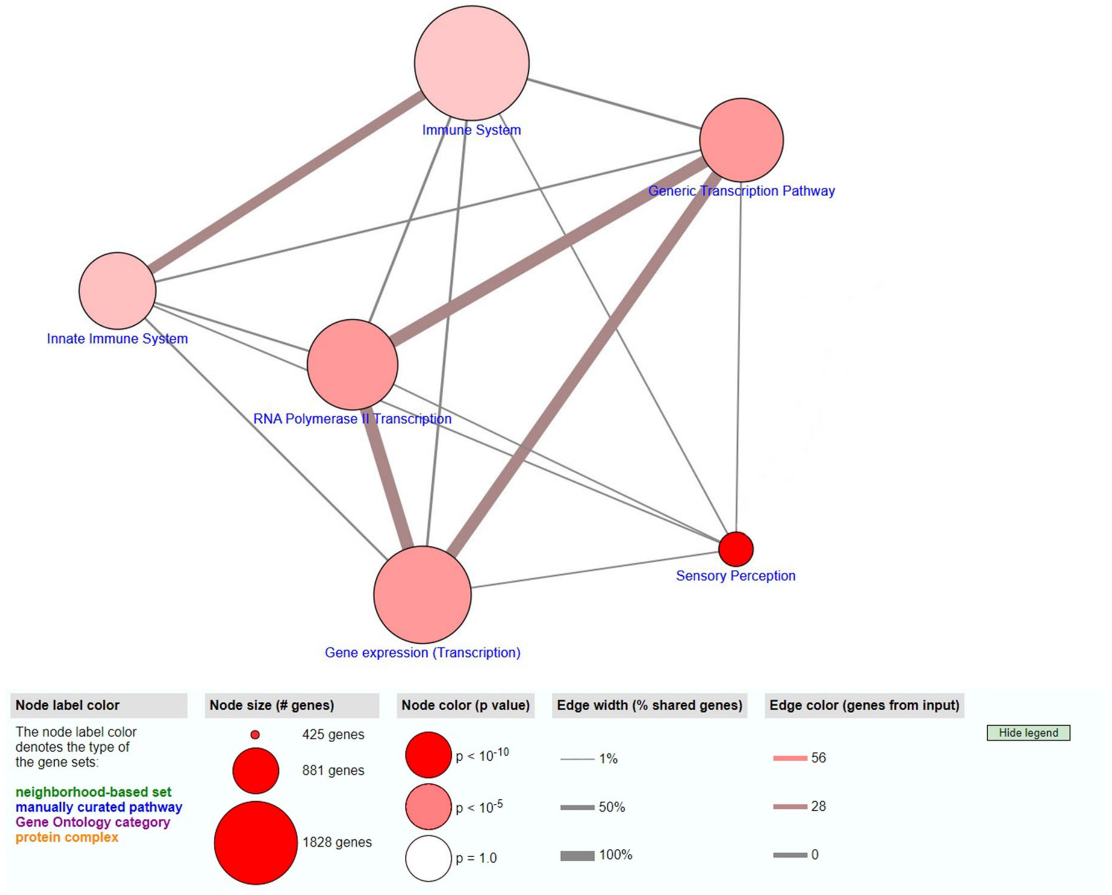

Supplementary Figure S1. The significantly enriched pathways of the top 400 DMGs between BrCa and healthy tissues based on REACTOME pathway analysis.

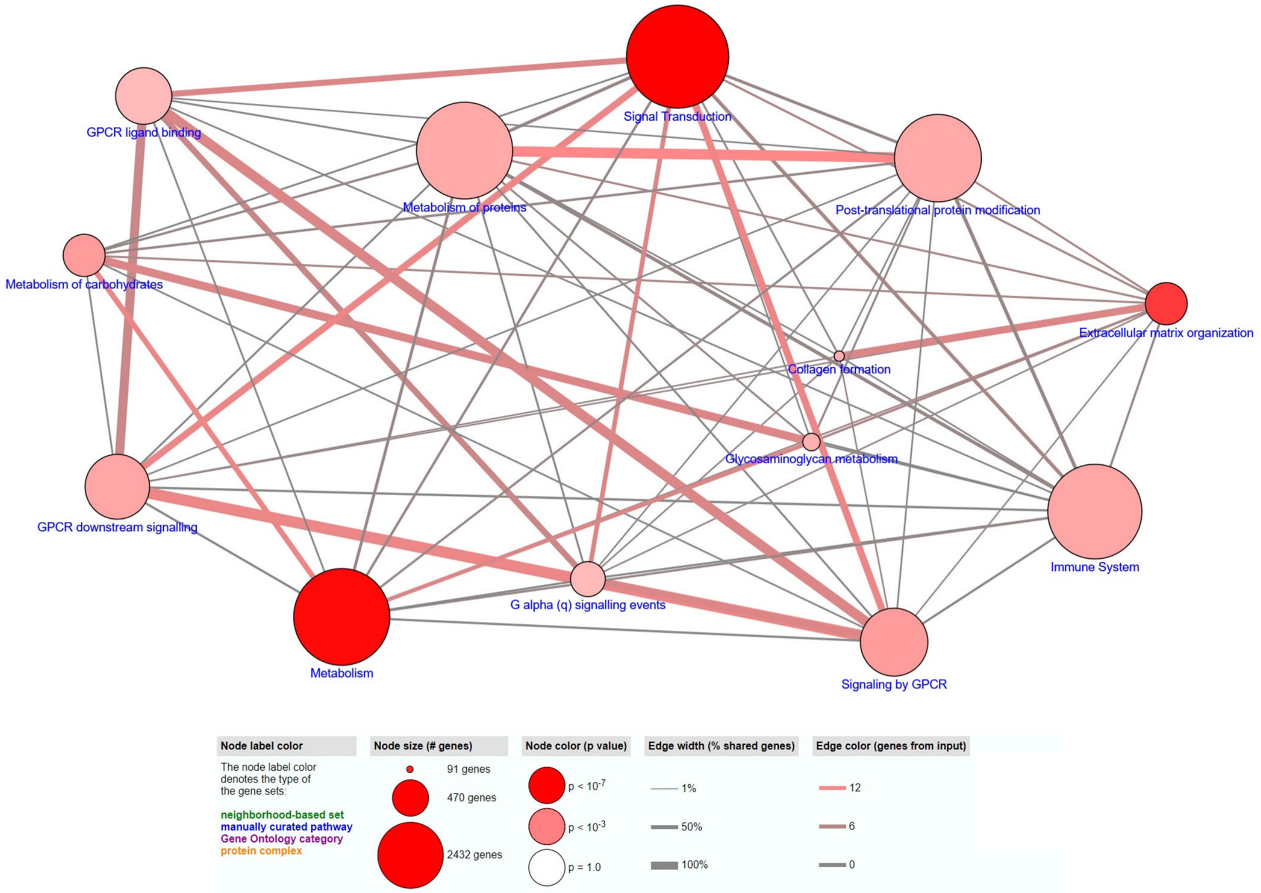

Supplementary Figure S2. The significantly enriched pathways of the top 400 DMGs between OA and healthy tissues based on REACTOME pathway analysis.

Supplementary Table S1. GEO studies and corresponding tissues/cell types used to retrieve methylomes for our analysis.

GEO Study ID	Tissue/ cell type	BrCa (number of samples)	OA (number of samples)	Pancreatic β-cell (number of samples)
GSE72245	Breast cancer	90	-	-
GSE72251	Breast cancer	98	-	-
GSE108576	Breast cancer	30	-	-
GSE63106	Preserved and Lesioned cartilage	-	62	-
GSE63695	OA Knee, Healthy and OA Hip cartilage	3	92	-
GSE73626	OA Knee, Healthy and OA hip cartilage	3	18	-
GSE65467	Articular chondrocytes	2	6	-
GSE122126	Pancreatic beta, acinar and alpha cells, Adipocytes, Hepatocytes	3	3	9
GSE48472	Whole blood, muscle, omentum, pancreas, subcutaneous adipose, spleen, buccal cells	35	35	2
GSE101764	Colon mucosa	3	3	1

GSE139404	Colon tissue	4	4	-
GSE103186	Gastric	3	3	-
GSE99553	Gastric mucosa	2	2	1
GSE97687	Oesophageal	4	4	1
GSE72872	Stomach, normal adjacent squamous	3	3	-
GSE38240	Prostate	-	2	-
GSE61278	Liver	3	3	1
GSE61446	Liver	2	2	-
GSE124367	Breast	6	4	1
GSE88883	Breast	6	4	-
GSE90060	Endometrium	4	4	-
GSE65820	Ovary fallopian Tube cells	3	3	1
GSE115797	Skin	4	4	1
GSE105018	Whole blood	10	10	-
GSE65638	Whole blood	10	10	2
GSE68777	Serum	10	10	2
GSE51057	Buffy coat	5	5	2
GSE130029	CD4+ T cells	3	3	-
GSE130030	CD8+ T cells	3	3	-
GSE49618	CD34+CD38- cells, Bone marrow CD19+ B cells, CD3+ T cells, Bone marrow CD3+ T cells, Bone marrow Promyelocytes, Monocytes, Polymorphonuclear granulocytes, Promyelocytes, Bone marrow Monocytes	16	16	-
GSE61107	Frontal cortex brain	3	3	-
GSE66351	Glia brain cells, Neuron cells	4	4	1
GSE59524	Visceral adipose	4	4	1
GSE61450	Subcutaneous adipose	3	3	1
GSE61453	Obese visceral adipose	3	3	-
GSE61452	Muscle	4	4	1
GSE60655	Vastus lateralis muscle	2	2	-
GSE85636	Vastus lateralis muscle	2	2	-
GSE62727	Left atrium	3	3	1
GSE75434	Superficial temporal artery	3	3	-
GSE84274	Ascending aorta	2	2	-
GSE66836	Lung tissue	3	3	-
GSE85566	Airway epithelial cells	3	3	1
GSE65163	Nasal epithelium	4	4	1

Abbreviations: BrCa Breast Cancer, OA Osteoarthritis

Supplementary Table S2. The list of the 400 top-ranking DMGs between BrCa and healthy tissues based on FDR.

Ensembl id	Gene symbol	Mean methylation difference	FDR	Methylation in BrCa tissues in relation to healthy tissues
ENSG00000170893	TRH	0.358	1.20E-91	Increase
ENSG00000170178	HOXD12	0.327	7.01E-88	Increase
ENSG00000151615	POU4F2	0.307	8.91E-88	Increase
ENSG00000255963	PPIAL4A	-0.264	8.00E-87	Decrease
ENSG00000145692	BHMT	0.315	3.31E-86	Increase
ENSG00000164736	SOX17	0.316	1.12E-84	Increase
ENSG00000187559	FOXD4L3	0.221	1.10E-82	Increase
ENSG00000170122	FOXD4	0.279	1.78E-82	Increase
ENSG00000117477	CCDC181	0.317	2.55E-81	Increase
ENSG00000186051	TAL2	-0.391	1.42E-80	Decrease
ENSG00000265843	LINC01029	-0.277	6.98E-80	Decrease
ENSG00000146618	FERD3L	0.347	3.00E-79	Increase
ENSG00000131068	DEFB118	-0.395	1.34E-78	Decrease
ENSG00000176435	CLEC14A	0.333	1.89E-78	Increase
ENSG00000186790	FOXE3	0.301	2.15E-78	Increase
ENSG00000177201	OR2T12	-0.279	5.38E-78	Decrease
ENSG00000177186	OR2M7	-0.412	2.71E-76	Decrease
ENSG00000125816	NKX2-4	0.267	3.54E-75	Increase
ENSG00000215474	SKOR2	0.217	1.71E-74	Increase
ENSG00000177233	OR2M1P	-0.285	4.12E-74	Decrease
ENSG00000188558	OR2G6	-0.355	6.91E-74	Decrease
ENSG00000121853	GHSR	0.215	7.00E-74	Increase
ENSG00000244349	HCG16	0.353	2.29E-73	Increase
ENSG00000135312	HTR1B	0.258	3.37E-73	Increase
ENSG00000187714	SLC18A3	0.262	1.99E-72	Increase
ENSG00000104722	NEFM	0.204	2.40E-72	Increase
ENSG00000227195	MIR663A	0.256	2.58E-72	Increase
ENSG00000235529	AGAP1-IT1	-0.306	3.05E-72	Decrease
ENSG00000143032	BARHL2	0.265	8.69E-72	Increase
ENSG00000240563	L1TD1	0.230	2.13E-71	Increase
ENSG00000236981	OR10G9	-0.309	2.21E-71	Decrease
ENSG00000169840	GSX1	0.284	2.76E-71	Increase
ENSG00000160791	CCR5	-0.336	3.38E-71	Decrease
ENSG00000176165	FOXG1	0.293	6.18E-71	Increase
ENSG00000148826	NKX6-2	0.271	7.69E-71	Increase
ENSG00000180053	NKX2-6	0.247	1.32E-70	Increase
ENSG00000182968	SOX1	0.261	7.37E-70	Increase
ENSG00000198327	HIST1H4F	0.297	1.25E-69	Increase
ENSG00000269067	ZNF728	0.147	1.47E-69	Increase
ENSG00000143631	FLG	-0.232	1.82E-69	Decrease

ENSG00000221957	KIR2DS4	-0.297	2.22E-69	Decrease
ENSG00000230749	MEIS1-AS2	0.371	4.21E-69	Increase
ENSG00000105131	EPHX3	0.282	4.77E-69	Increase
ENSG00000189181	OR14I1	-0.321	5.06E-69	Decrease
ENSG00000228198	OR2M3	-0.386	6.57E-69	Decrease
ENSG00000207933	MIR9-1	0.325	1.30E-68	Increase
ENSG00000143954	REG3G	-0.287	1.91E-68	Decrease
ENSG00000207994	MIR100	-0.328	8.32E-68	Decrease
ENSG00000184155	OR10J5	-0.336	2.13E-67	Decrease
ENSG00000196171	OR6K2	-0.251	6.17E-67	Decrease
ENSG00000196266	OR10J3	-0.332	6.85E-67	Decrease
ENSG00000178919	FOXE1	0.309	1.85E-66	Increase
ENSG00000197532	OR6Y1	-0.300	3.32E-66	Decrease
ENSG00000198601	OR2M2	-0.341	7.44E-66	Decrease
ENSG00000219891	ZSCAN12P1	0.274	1.30E-65	Increase
ENSG00000166118	SPATA19	-0.293	1.31E-65	Decrease
ENSG00000261614	YBX3P1	0.221	1.45E-65	Increase
ENSG00000137265	IRF4	0.098	1.80E-65	Increase
ENSG00000006128	TAC1	0.254	1.84E-65	Increase
ENSG00000165588	OTX2	0.233	3.38E-65	Increase
ENSG00000166342	NETO1	0.189	3.57E-65	Increase
ENSG00000183566	BPIFA4P	-0.293	8.76E-65	Decrease
ENSG00000092607	TBX15	0.256	1.03E-64	Increase
ENSG00000001626	CFTR	0.227	2.07E-64	Increase
ENSG00000267365	KCNJ2-AS1	0.279	2.27E-64	Increase
ENSG00000162992	NEUROD1	0.266	2.47E-64	Increase
ENSG00000181195	PENK	0.139	3.31E-64	Increase
ENSG00000196539	OR2T3	-0.389	4.26E-64	Decrease
ENSG00000172016	REG3A	-0.305	5.75E-64	Decrease
ENSG00000268751	SCGB1B2P	0.256	5.81E-64	Increase
ENSG00000100285	NEFH	0.226	3.58E-63	Increase
ENSG00000162618	ELTD1	0.297	4.40E-63	Increase
ENSG00000125533	BHLHE23	0.265	4.55E-63	Increase
ENSG00000229415	SFTA3	0.226	7.03E-63	Increase
ENSG00000187140	FOXD3	0.300	1.14E-62	Increase
ENSG00000173080	RXFP4	-0.324	2.03E-62	Decrease
ENSG00000132703	APCS	-0.338	3.05E-62	Decrease
ENSG00000109132	PHOX2B	0.262	4.92E-62	Increase
ENSG00000215784	FAM72D	-0.302	6.54E-62	Decrease
ENSG00000172023	REG1B	-0.264	6.74E-62	Decrease
ENSG00000180068	OR3A4P	-0.253	7.47E-62	Decrease
ENSG00000248522	SBF1P1	-0.223	9.82E-62	Decrease

ENSG00000198028	ZNF560	0.113	1.04E-61	Increase
ENSG00000105371	ICAM4	0.252	1.05E-61	Increase
ENSG00000199077	MIR129-2	0.302	1.59E-61	Increase
ENSG00000256128	LINC00944	-0.267	2.27E-61	Decrease
ENSG00000162727	OR2M5	-0.293	5.27E-61	Decrease
ENSG00000171540	OTP	0.229	6.03E-61	Increase
ENSG00000101438	SLC32A1	0.216	7.08E-61	Increase
ENSG00000173401	GLIPR1L1	0.272	7.20E-61	Increase
ENSG00000147465	STAR	0.205	8.07E-61	Increase
ENSG00000128709	HOXD9	0.284	8.94E-61	Increase
ENSG00000184492	FOXD4L1	0.198	1.00E-60	Increase
ENSG00000168995	SIGLEC7	-0.245	1.22E-60	Decrease
ENSG00000177468	OLIG3	0.279	1.82E-60	Increase
ENSG00000174279	EVX2	0.248	4.81E-60	Increase
ENSG00000180483	DEFB119	-0.297	5.16E-60	Decrease
ENSG00000223638	RFPL4A	-0.273	7.78E-60	Decrease
ENSG00000187701	OR2T27	-0.245	9.36E-60	Decrease
ENSG00000168875	SOX14	0.209	1.13E-59	Increase
ENSG00000234445	FGF14-AS1	-0.275	1.25E-59	Decrease
ENSG00000229590	MSX2P1	0.211	1.25E-59	Increase
ENSG00000232709	MARK2P9	0.237	1.63E-59	Increase
ENSG00000159182	PRAC1	0.330	2.59E-59	Increase
ENSG00000129596	CDO1	0.228	2.96E-59	Increase
ENSG00000199004	MIR21	-0.380	5.29E-59	Decrease
ENSG00000156150	ALX3	0.235	5.79E-59	Increase
ENSG00000109851	DBX1	0.250	1.03E-58	Increase
ENSG00000122584	NXPH1	0.235	1.06E-58	Increase
ENSG00000167769	ACER1	-0.214	1.50E-58	Decrease
ENSG00000100987	VSX1	0.209	1.52E-58	Increase
ENSG00000229240	LINC00710	-0.243	2.30E-58	Decrease
ENSG00000183130	OR2T11	-0.254	2.40E-58	Decrease
ENSG00000234749	FAM90A21P	-0.163	3.20E-58	Decrease
ENSG00000109158	GABRA4	0.151	3.64E-58	Increase
ENSG00000177875	C12orf68	0.201	3.91E-58	Increase
ENSG00000260549	MT1L	0.353	3.91E-58	Increase
ENSG00000186572	DEFB107A	-0.352	6.37E-58	Decrease
ENSG00000128713	HOXD11	0.256	7.02E-58	Increase
ENSG00000206474	OR10C1	-0.201	8.77E-58	Decrease
ENSG00000207695	MIR184	0.363	1.21E-57	Increase
ENSG00000167968	DNASE1L2	0.251	1.33E-57	Increase
ENSG00000172146	OR1A1	-0.282	1.71E-57	Decrease
ENSG00000184302	SIX6	0.269	1.82E-57	Increase

ENSG00000180708	OR10K2	-0.335	2.38E-57	Decrease
ENSG00000261787	TCF24	0.232	3.44E-57	Increase
ENSG00000177511	ST8SIA3	0.150	4.44E-57	Increase
ENSG00000123977	DAW1	0.194	8.18E-57	Increase
ENSG00000112238	PRDM13	0.212	8.37E-57	Increase
ENSG00000054803	CBLN4	0.162	8.62E-57	Increase
ENSG00000088882	CPXM1	0.208	8.75E-57	Increase
ENSG00000153266	FEZF2	0.219	8.75E-57	Increase
ENSG00000124557	BTN1A1	0.177	9.66E-57	Increase
ENSG00000160882	CYP11B1	-0.232	1.58E-56	Decrease
ENSG00000132693	CRP	-0.259	1.63E-56	Decrease
ENSG00000101017	CD40	0.129	1.87E-56	Increase
ENSG00000198558	HIST1H4L	0.225	1.94E-56	Increase
ENSG00000006116	CACNG3	-0.059	1.95E-56	Decrease
ENSG00000235884	LINC00941	0.168	2.91E-56	Increase
ENSG00000150337	FCGR1A	-0.165	5.37E-56	Decrease
ENSG00000207604	MIR206	-0.302	5.68E-56	Decrease
ENSG00000168267	PTF1A	0.212	5.82E-56	Increase
ENSG00000204491	PRAMEF18	-0.210	6.11E-56	Decrease
ENSG00000234456	MAGI2-AS3	0.231	7.38E-56	Increase
ENSG00000122254	HS3ST2	-0.051	8.39E-56	Decrease
ENSG00000178860	MSC	0.241	1.19E-55	Increase
ENSG00000101405	OXT	0.297	1.25E-55	Increase
ENSG00000152760	TCTEX1D1	0.196	1.28E-55	Increase
ENSG00000226995	LINC00658	-0.212	1.33E-55	Decrease
ENSG00000091010	POU4F3	0.223	3.25E-55	Increase
ENSG00000179172	HNRNPCL1	-0.170	4.48E-55	Decrease
ENSG00000187987	ZSCAN23	0.122	4.65E-55	Increase
ENSG00000130487	KLHDC7B	0.233	6.09E-55	Increase
ENSG00000183807	FAM162B	0.159	6.40E-55	Increase
ENSG00000179008	C14orf39	0.237	6.40E-55	Increase
ENSG00000166573	GALR1	0.249	7.91E-55	Increase
ENSG00000198104	OR2T6	-0.327	1.02E-54	Decrease
ENSG00000170929	OR1M1	-0.217	1.12E-54	Decrease
ENSG00000267470	ZNF571-AS1	0.174	1.80E-54	Increase
ENSG00000122859	NEUROG3	0.198	2.90E-54	Increase
ENSG00000125798	FOXA2	0.211	3.06E-54	Increase
ENSG00000163554	SPTA1	-0.291	3.78E-54	Decrease
ENSG00000204793	FOXD4L6	0.246	3.87E-54	Increase
ENSG00000212544	RNVU1-19	0.069	4.22E-54	Increase
ENSG00000101463	SYNDIG1	0.150	5.80E-54	Increase
ENSG00000178235	SLITRK1	0.221	5.94E-54	Increase

ENSG00000188816	HMX2	0.241	5.96E-54	Increase
ENSG00000112837	TBX18	0.213	8.04E-54	Increase
ENSG00000134398	ERN2	0.156	1.18E-53	Increase
ENSG00000163081	CCDC140	0.239	1.57E-53	Increase
ENSG00000179909	ZNF154	0.263	1.95E-53	Increase
ENSG00000214787	MS4A4E	-0.094	2.13E-53	Decrease
ENSG00000112333	NR2E1	0.247	2.22E-53	Increase
ENSG00000128310	GALR3	0.220	2.31E-53	Increase
ENSG00000124900	TRIM51	-0.221	2.59E-53	Decrease
ENSG00000243197	TUSC7	-0.255	4.55E-53	Decrease
ENSG00000116996	ZP4	-0.298	5.55E-53	Decrease
ENSG00000269964	MEI4	-0.086	5.81E-53	Decrease
ENSG00000196834	POTEI	-0.167	6.66E-53	Decrease
ENSG00000189238	LINC00943	-0.110	7.37E-53	Decrease
ENSG00000166884	OR4D6	-0.262	7.43E-53	Decrease
ENSG00000168787	OR12D2	-0.236	9.18E-53	Decrease
ENSG00000237380	HOXD-AS2	0.254	9.47E-53	Increase
ENSG00000240990	HOXA11-AS	0.235	9.90E-53	Increase
ENSG00000162624	LHX8	0.230	1.13E-52	Increase
ENSG00000164379	FOXQ1	0.228	1.22E-52	Increase
ENSG00000179242	CDH4	-0.187	1.24E-52	Decrease
ENSG00000177535	OR2B11	-0.211	1.43E-52	Decrease
ENSG00000177462	OR2T8	-0.178	1.76E-52	Decrease
ENSG00000171180	OR2M4	-0.249	1.92E-52	Decrease
ENSG00000158488	CD1E	-0.276	2.04E-52	Decrease
ENSG00000253250	C8orf88	0.098	2.14E-52	Increase
ENSG00000112246	SIM1	0.256	3.37E-52	Increase
ENSG00000169877	AHSP	-0.301	3.57E-52	Decrease
ENSG00000078399	HOXA9	0.246	3.57E-52	Increase
ENSG00000196184	OR10J1	-0.274	3.57E-52	Decrease
ENSG00000152192	POU4F1	0.252	3.85E-52	Increase
ENSG00000059804	SLC2A3	0.207	5.04E-52	Increase
ENSG00000106038	EVX1	0.237	5.12E-52	Increase
ENSG00000124935	SCGB1D2	-0.210	5.47E-52	Decrease
ENSG00000188069	OR51F1	-0.296	6.06E-52	Decrease
ENSG00000157005	SST	0.157	6.10E-52	Increase
ENSG00000158887	MPZ	0.184	6.40E-52	Increase
ENSG00000102924	CBLN1	0.226	6.64E-52	Increase
ENSG00000181965	NEUROG1	0.226	7.13E-52	Increase
ENSG00000116726	PRAMEF12	-0.204	7.53E-52	Decrease
ENSG00000181234	TMEM132C	-0.144	9.51E-52	Decrease
ENSG00000143632	ACTA1	0.212	1.09E-51	Increase

ENSG00000165556	CDX2	0.216	1.25E-51	Increase
ENSG00000107807	TLX1	0.238	1.39E-51	Increase
ENSG00000166363	OR10A5	-0.264	1.48E-51	Decrease
ENSG00000212724	KRTAP2-3	0.270	2.01E-51	Increase
ENSG00000205358	MT1H	0.158	2.78E-51	Increase
ENSG00000125820	NKX2-2	0.256	3.14E-51	Increase
ENSG00000175426	PCSK1	0.202	4.81E-51	Increase
ENSG00000186440	OR6P1	-0.329	5.44E-51	Decrease
ENSG00000182255	KCNA4	-0.007	6.12E-51	Decrease
ENSG00000122585	NPY	0.148	1.03E-50	Increase
ENSG00000139874	SSTR1	0.223	1.04E-50	Increase
ENSG00000111046	MYF6	0.251	1.07E-50	Increase
ENSG00000173157	ADAMTS20	0.081	1.15E-50	Increase
ENSG00000147613	PSKH2	0.176	1.23E-50	Increase
ENSG00000135577	NMBR	0.200	2.03E-50	Increase
ENSG00000182901	RGS7	-0.161	2.08E-50	Decrease
ENSG00000100918	REC8	0.278	2.08E-50	Increase
ENSG00000166148	AVPR1A	0.174	2.32E-50	Increase
ENSG00000186306	OR10T2	-0.323	2.99E-50	Decrease
ENSG00000181418	DDN	0.216	3.65E-50	Increase
ENSG00000204548	DEFB121	-0.214	4.02E-50	Decrease
ENSG00000206422	LRRC30	-0.244	4.55E-50	Decrease
ENSG00000111981	ULBP1	0.128	4.75E-50	Increase
ENSG00000235026	DPP10-AS1	0.320	5.13E-50	Increase
ENSG00000198967	OR10Z1	-0.292	6.24E-50	Decrease
ENSG00000225556	C2CD4D	0.242	6.87E-50	Increase
ENSG00000180340	FZD2	0.178	7.72E-50	Increase
ENSG00000147596	PRDM14	0.167	8.28E-50	Increase
ENSG00000176635	HORMAD2	0.214	1.05E-49	Increase
ENSG00000208038	MIR492	-0.163	1.12E-49	Decrease
ENSG00000136535	TBR1	0.243	1.20E-49	Increase
ENSG00000125492	BARHL1	0.210	1.36E-49	Increase
ENSG00000171014	OR4D5	-0.259	1.55E-49	Decrease
ENSG00000057149	SERPINB3	-0.219	1.69E-49	Decrease
ENSG00000156959	LHFPL4	0.124	1.69E-49	Increase
ENSG00000169717	ACTRT2	-0.177	1.72E-49	Decrease
ENSG00000184937	WT1	0.214	1.75E-49	Increase
ENSG00000151952	TMEM132D	-0.193	2.23E-49	Decrease
ENSG00000115386	REG1A	-0.239	2.24E-49	Decrease
ENSG00000060709	RIMBP2	-0.195	2.52E-49	Decrease
ENSG00000204694	OR11A1	-0.212	2.53E-49	Decrease
ENSG00000205457	TP53TG3C	-0.246	2.81E-49	Decrease

ENSG00000163207	IVL	-0.241	3.76E-49	Decrease
ENSG00000170866	LILRA3	-0.178	3.86E-49	Decrease
ENSG00000181963	OR52K2	-0.223	4.19E-49	Decrease
ENSG00000168356	SCN11A	-0.200	4.19E-49	Decrease
ENSG00000207613	MIR181C	-0.206	4.33E-49	Decrease
ENSG00000198339	HIST1H4I	0.313	4.62E-49	Increase
ENSG00000111241	FGF6	-0.189	5.24E-49	Decrease
ENSG00000197403	OR6N1	-0.262	6.44E-49	Decrease
ENSG00000233429	HOTAIRM1	0.232	6.44E-49	Increase
ENSG00000166501	PRKCB	-0.094	6.56E-49	Decrease
ENSG00000118946	PCDH17	0.147	6.61E-49	Increase
ENSG00000196936	OR2L8	-0.192	7.35E-49	Decrease
ENSG00000226790	HNRNPA3P1	-0.244	8.54E-49	Decrease
ENSG00000123700	KCNJ2	0.028	9.31E-49	Increase
ENSG00000187135	VSTM2B	0.162	9.65E-49	Increase
ENSG00000206172	HBA1	0.182	1.04E-48	Increase
ENSG00000143355	LHX9	0.228	1.08E-48	Increase
ENSG00000109832	DDX25	0.156	1.18E-48	Increase
ENSG00000110484	SCGB2A2	-0.213	1.29E-48	Decrease
ENSG00000153666	GOLGA8I	-0.157	1.33E-48	Decrease
ENSG00000145248	SLC10A4	0.197	1.38E-48	Increase
ENSG00000260102	LINC01070	-0.274	1.71E-48	Decrease
ENSG00000163202	LCE3D	-0.239	1.76E-48	Decrease
ENSG00000159961	OR3A3	-0.183	1.96E-48	Decrease
ENSG00000105954	NPVF	-0.245	2.18E-48	Decrease
ENSG00000198807	PAX9	0.227	3.10E-48	Increase
ENSG00000173262	SLC2A14	0.111	3.34E-48	Increase
ENSG00000267339	LINC00906	-0.259	4.00E-48	Decrease
ENSG00000142700	DMRTA2	0.229	4.06E-48	Increase
ENSG00000163623	NKX6-1	0.217	4.41E-48	Increase
ENSG00000176937	OR52R1	-0.231	4.73E-48	Decrease
ENSG00000132554	RGS22	0.156	4.78E-48	Increase
ENSG00000204695	OR14J1	-0.248	5.05E-48	Decrease
ENSG00000197838	CYP2A13	0.173	5.15E-48	Increase
ENSG00000188536	HBA2	0.139	5.47E-48	Increase
ENSG00000077080	ACTL6B	0.152	5.67E-48	Increase
ENSG00000172156	CCL11	-0.273	5.81E-48	Decrease
ENSG00000204687	MAS1L	-0.249	5.83E-48	Decrease
ENSG00000127529	OR7C2	-0.227	6.64E-48	Decrease
ENSG00000200089	SNORD114-31	-0.227	7.12E-48	Decrease
ENSG00000006788	MYH13	-0.204	7.39E-48	Decrease
ENSG00000008197	TFAP2D	0.178	7.66E-48	Increase

ENSG00000183598	HIST2H3D	0.175	8.27E-48	Increase
ENSG00000165443	PHYHIPL	0.154	8.31E-48	Increase
ENSG00000170782	OR10A4	-0.251	9.19E-48	Decrease
ENSG00000165462	PHOX2A	0.187	9.42E-48	Increase
ENSG00000221882	OR3A2	-0.187	1.19E-47	Decrease
ENSG00000170923	OR7G2	-0.247	1.70E-47	Decrease
ENSG00000264424	MYH4	-0.264	1.76E-47	Decrease
ENSG00000197915	HRNR	-0.152	2.12E-47	Decrease
ENSG00000181939	OR4C15	-0.257	2.13E-47	Decrease
ENSG00000128710	HOXD10	0.220	2.27E-47	Increase
ENSG00000101292	PROKR2	-0.107	2.39E-47	Decrease
ENSG00000178645	C10orf53	-0.038	3.01E-47	Decrease
ENSG00000205176	REXO1L1P	-0.159	3.51E-47	Decrease
ENSG00000227888	FAM66A	-0.167	3.80E-47	Decrease
ENSG00000013293	SLC7A14	0.116	4.40E-47	Increase
ENSG00000125285	SOX21	0.209	4.50E-47	Increase
ENSG00000204519	ZNF551	0.202	4.74E-47	Increase
ENSG00000172289	OR10V1	-0.271	5.42E-47	Decrease
ENSG00000148798	INA	0.167	6.29E-47	Increase
ENSG00000164093	PITX2	0.231	6.81E-47	Increase
ENSG00000184530	C6orf58	0.155	7.04E-47	Increase
ENSG00000260738	LINC00554	0.202	8.01E-47	Increase
ENSG00000134762	DSC3	0.227	8.43E-47	Increase
ENSG00000152034	MCHR2	0.134	8.95E-47	Increase
ENSG00000176925	OR51F2	-0.315	9.88E-47	Decrease
ENSG00000183242	WT1-AS	0.206	9.96E-47	Increase
ENSG00000138769	CDKL2	0.190	1.01E-46	Increase
ENSG00000198155	ZNF876P	0.182	1.02E-46	Increase
ENSG00000206579	XKR4	-0.140	1.05E-46	Decrease
ENSG00000103449	SALL1	0.099	1.07E-46	Increase
ENSG00000137463	MGARP	0.195	1.08E-46	Increase
ENSG00000229315	MCHR2-AS1	0.180	1.22E-46	Increase
ENSG00000198185	ZNF334	0.143	1.29E-46	Increase
ENSG00000109061	MYH1	-0.217	1.36E-46	Decrease
ENSG00000171794	UTF1	0.231	1.37E-46	Increase
ENSG00000253302	STAU2-AS1	-0.303	1.54E-46	Decrease
ENSG00000151948	GLT1D1	-0.092	1.66E-46	Decrease
ENSG00000119973	PRLHR	0.175	1.74E-46	Increase
ENSG00000169427	KCNK9	-0.086	1.80E-46	Decrease
ENSG00000170255	MRGPRX1	-0.189	2.02E-46	Decrease
ENSG00000183770	FOXL2	0.234	2.05E-46	Increase
ENSG00000253661	ZFHX4-AS1	0.195	2.37E-46	Increase

ENSG00000130226	DPP6	-0.088	2.39E-46	Decrease
ENSG00000231824	C18orf42	0.071	2.68E-46	Increase
ENSG00000198885	ITPRIPL1	0.153	2.69E-46	Increase
ENSG00000136110	LECT1	0.161	3.66E-46	Increase
ENSG00000104435	STMN2	0.179	3.70E-46	Increase
ENSG00000221931	OR6X1	-0.225	3.80E-46	Decrease
ENSG00000188859	FAM78B	-0.062	3.95E-46	Decrease
ENSG00000144290	SLC4A10	0.175	4.52E-46	Increase
ENSG00000112539	C6orf118	-0.113	5.33E-46	Decrease
ENSG00000145020	AMT	0.195	5.77E-46	Increase
ENSG00000244020	MT1HL1	-0.180	6.00E-46	Decrease
ENSG00000233988	ADAM6	-0.171	6.23E-46	Decrease
ENSG00000115297	TLX2	0.211	6.68E-46	Increase
ENSG00000112499	SLC22A2	-0.201	7.15E-46	Decrease
ENSG00000100276	RASL10A	0.179	8.23E-46	Increase
ENSG00000128714	HOXD13	0.245	9.32E-46	Increase
ENSG00000204983	PRSS1	-0.241	9.49E-46	Decrease
ENSG00000182103	FAM181B	0.233	1.06E-45	Increase
ENSG00000205879	FAM90A2P	-0.126	1.23E-45	Decrease
ENSG00000173530	TNFRSF10D	0.126	1.26E-45	Increase
ENSG00000243729	OR5V1	-0.215	2.00E-45	Decrease
ENSG00000171053	PATE1	-0.206	2.09E-45	Decrease
ENSG00000175040	CHST2	0.195	2.11E-45	Increase
ENSG00000148704	VAX1	0.220	2.11E-45	Increase
ENSG00000176200	OR4D11	-0.232	2.35E-45	Decrease
ENSG00000262096	PCDHB19P	0.214	3.16E-45	Increase
ENSG00000196341	OR8D1	-0.192	3.30E-45	Decrease
ENSG00000225725	FAM66E	-0.187	3.44E-45	Decrease
ENSG00000198914	POU3F3	0.216	3.68E-45	Increase
ENSG00000227262	HCG4B	0.233	3.72E-45	Increase
ENSG00000164532	TBX20	0.222	4.14E-45	Increase
ENSG00000230223	ATXN8OS	0.208	5.17E-45	Increase
ENSG00000183783	KCTD8	0.093	5.69E-45	Increase
ENSG00000158485	CD1B	-0.313	6.09E-45	Decrease
ENSG00000207816	MIR124-2	0.320	6.44E-45	Increase
ENSG00000207742	MIR382	-0.256	8.79E-45	Decrease
ENSG00000164438	TLX3	0.217	9.19E-45	Increase
ENSG00000136014	USP44	0.189	1.00E-44	Increase
ENSG00000226288	OR52I2	-0.209	1.11E-44	Decrease
ENSG00000170128	GPR25	0.206	1.12E-44	Increase
ENSG00000172154	OR8I2	-0.197	1.17E-44	Decrease
ENSG00000204700	OR2J2	-0.205	1.21E-44	Decrease

ENSG00000119125	GDA	0.027	1.43E-44	Increase
ENSG00000164853	UNCX	0.181	1.53E-44	Increase
ENSG00000166482	MFAP4	0.222	1.53E-44	Increase
ENSG00000185274	WBSCR17	-0.106	1.62E-44	Decrease
ENSG00000164778	EN2	0.218	1.79E-44	Increase
ENSG00000119547	ONECUT2	0.168	1.89E-44	Increase
ENSG00000186400	OR10X1	-0.308	1.99E-44	Decrease
ENSG00000078328	RBFOX1	-0.123	2.01E-44	Decrease
ENSG00000259282	SPATA8-AS1	-0.211	2.06E-44	Decrease
ENSG00000185594	SPATA8	-0.224	2.43E-44	Decrease
ENSG00000108753	HNF1B	0.143	2.53E-44	Increase
ENSG00000156284	CLDN8	-0.232	2.79E-44	Decrease
ENSG00000139515	PDX1	0.190	3.81E-44	Increase
ENSG00000233780	HNRNPA1P30	0.196	4.25E-44	Increase
ENSG00000189013	KIR2DL4	-0.191	4.40E-44	Decrease
ENSG00000234467	SLC25A1P2	-0.158	4.80E-44	Decrease
ENSG00000229419	RALGAPA1P	-0.178	4.88E-44	Decrease
ENSG00000206072	SERPINB11	-0.200	4.88E-44	Decrease
ENSG00000183640	KRTAP8-1	-0.240	5.62E-44	Decrease
ENSG00000168060	NAALADL1	0.199	5.75E-44	Increase
ENSG00000174948	GPR149	0.176	5.92E-44	Increase
ENSG00000123364	HOXC13	0.154	6.19E-44	Increase
ENSG00000259571	BLID	-0.299	6.44E-44	Decrease
ENSG00000197790	OR52M1	-0.268	6.68E-44	Decrease

Supplementary Table S3. The list of the 400 top-ranking DMGs between OA and healthy tissues.

Ensembl id	Gene symbol	Mean methylation difference	FDR	Methylation in OA in relation to healthy tissues
ENSG00000212232	SNORD17	0.407	6.49E-77	Decrease
ENSG00000143185	XCL2	0.291	4.65E-69	Decrease
ENSG00000203864	C1orf137	0.528	1.93E-68	Decrease
ENSG00000143196	DPT	0.531	2.79E-68	Decrease
ENSG00000207500	SNORD102	0.605	5.19E-68	Decrease
ENSG00000222246	MIR1471	0.308	1.19E-66	Decrease
ENSG00000181541	MAB21L2	0.498	1.02E-65	Decrease
ENSG00000167874	TMEM88	0.309	1.14E-65	Decrease
ENSG00000129009	ISLR	0.403	1.41E-65	Decrease
ENSG00000170688	OR5E1P	0.388	2.49E-65	Decrease
ENSG00000176678	FOXL1	-0.362	1.32E-63	Increase
ENSG00000199104	MIR346	0.513	1.37E-63	Decrease
ENSG00000226746	SMCR5	0.509	4.37E-63	Decrease
ENSG00000231764	DLX6-AS1	-0.235	2.76E-61	Increase
ENSG00000204930	FAM221B	0.624	3.38E-61	Decrease

ENSG00000260785	CASC17	0.549	3.83E-61	Decrease
ENSG00000122176	FMOD	0.296	2.00E-60	Decrease
ENSG00000204193	TXNDC8	0.577	6.82E-59	Decrease
ENSG00000211520	MIR216B	0.465	1.65E-58	Decrease
ENSG00000170166	HOXD4	-0.377	2.08E-58	Increase
ENSG00000223392	CLDN10-AS1	0.466	2.44E-58	Decrease
ENSG00000207751	MIR130B	0.352	4.99E-58	Decrease
ENSG00000118245	TNP1	0.251	1.01E-57	Decrease
ENSG00000180332	KCTD4	0.479	1.04E-57	Decrease
ENSG00000207752	MIR199A1	0.577	1.40E-57	Decrease
ENSG00000149968	MMP3	0.303	2.28E-57	Decrease
ENSG00000137976	DNASE2B	0.260	2.85E-57	Decrease
ENSG00000198373	WWP2	0.369	2.93E-57	Decrease
ENSG00000230630	DNM3OS	0.564	3.29E-57	Decrease
ENSG00000113520	IL4	0.522	1.34E-56	Decrease
ENSG00000207744	MIR10B	-0.365	1.90E-56	Increase
ENSG00000236520	GPC6-AS1	0.549	2.07E-56	Decrease
ENSG00000255399	TBX5-AS1	-0.378	3.23E-56	Increase
ENSG00000143127	ITGA10	0.408	3.67E-56	Decrease
ENSG00000175513	TSGA10IP	0.225	3.74E-56	Decrease
ENSG00000179766	ATP8B5P	0.323	1.08E-55	Decrease
ENSG00000199157	MIR208A	0.294	1.74E-55	Decrease
ENSG00000215183	MSMP	0.363	1.94E-55	Decrease
ENSG00000229671	LINC01150	0.523	6.16E-55	Decrease
ENSG00000207574	MIR661	0.268	2.16E-54	Decrease
ENSG00000164385	C6orf195	0.295	5.66E-54	Decrease
ENSG00000111341	MGP	0.278	9.23E-54	Decrease
ENSG00000161281	COX7A1	0.392	1.81E-53	Decrease
ENSG00000188783	PRELP	0.141	2.63E-53	Decrease
ENSG00000261857	MIA	0.311	3.35E-53	Decrease
ENSG00000188763	FZD9	0.307	2.15E-52	Decrease
ENSG00000257315	ZBED6	0.225	2.36E-52	Decrease
ENSG00000106038	EVX1	-0.231	2.69E-52	Increase
ENSG00000145681	HAPLN1	0.314	4.69E-52	Decrease
ENSG00000237941	KCNQ1DN	0.298	6.14E-52	Decrease
ENSG00000124216	SNAI1	0.308	9.82E-52	Decrease
ENSG00000082196	C1QTNF3	0.325	2.22E-51	Decrease
ENSG00000251493	FOXD1	-0.229	3.66E-51	Increase
ENSG00000188257	PLA2G2A	0.203	3.67E-51	Decrease
ENSG00000221836	OR2A5	0.336	6.02E-51	Decrease
ENSG00000141469	SLC14A1	0.327	8.69E-51	Decrease
ENSG00000250433	CLSTN2-AS1	0.324	9.40E-51	Decrease
ENSG00000106952	TNFSF8	0.152	1.52E-50	Decrease
ENSG00000099984	GSTT2	0.128	2.36E-50	Decrease
ENSG00000123388	HOXC11	-0.105	3.31E-50	Increase

ENSG00000233840	PCDH9-AS4	0.202	3.36E-50	Decrease
ENSG00000037965	HOXC8	-0.214	3.45E-50	Increase
ENSG00000213221	DNLZ	0.287	3.49E-50	Decrease
ENSG00000159899	NPR2	0.241	5.69E-50	Decrease
ENSG00000207949	MIR214	0.420	6.17E-50	Decrease
ENSG00000235621	LINC00494	0.474	7.94E-50	Decrease
ENSG00000159263	SIM2	-0.250	8.85E-50	Increase
ENSG00000143545	RAB13	0.260	9.16E-50	Decrease
ENSG00000182685	BRICD5	0.206	9.49E-50	Decrease
ENSG00000243766	HOTTIP	-0.181	1.06E-49	Increase
ENSG00000105880	DLX5	-0.193	2.41E-49	Increase
ENSG00000207691	MIR183	0.218	4.43E-49	Decrease
ENSG00000176971	FIBIN	0.187	5.15E-49	Decrease
ENSG00000251209	LINC00923	0.209	8.40E-49	Decrease
ENSG00000168779	SHOX2	-0.147	8.81E-49	Increase
ENSG00000139574	NPFF	0.163	8.98E-49	Decrease
ENSG00000207780	MIR648	-0.399	2.83E-48	Increase
ENSG00000173431	RNASE8	0.139	3.50E-48	Decrease
ENSG00000198973	MIR375	-0.206	3.74E-48	Increase
ENSG00000204361	NXPE2	0.264	8.20E-48	Decrease
ENSG00000143333	RGS16	0.243	1.45E-47	Decrease
ENSG00000096395	MLN	0.204	1.60E-47	Decrease
ENSG00000197445	C16orf47	0.439	2.45E-47	Decrease
ENSG00000221035	MIR486	0.212	2.56E-47	Decrease
ENSG00000185186	LINC00313	0.237	2.72E-47	Decrease
ENSG00000208024	MIR199A2	0.557	3.55E-47	Decrease
ENSG00000099869	IGF2-AS	-0.242	1.44E-46	Increase
ENSG00000162733	DDR2	0.363	2.66E-46	Decrease
ENSG00000166292	TMEM100	0.197	4.65E-46	Decrease
ENSG00000199015	MIR377	0.281	4.91E-46	Decrease
ENSG00000170454	KRT75	0.203	5.53E-46	Decrease
ENSG00000186860	KRTAP17-1	0.224	6.45E-46	Decrease
ENSG00000138615	CILP	0.282	6.55E-46	Decrease
ENSG00000106333	PCOLCE	0.044	6.98E-46	Decrease
ENSG00000149201	CCDC81	0.127	1.03E-45	Decrease
ENSG00000133800	LYVE1	0.199	1.06E-45	Decrease
ENSG00000182896	TMEM95	0.231	1.96E-45	Decrease
ENSG00000198353	HOXC4	-0.194	2.78E-45	Increase
ENSG00000155918	RAET1L	0.088	4.40E-45	Decrease
ENSG00000130592	LSP1	0.256	5.44E-45	Decrease
ENSG00000212402	SNORA74B	0.367	8.68E-45	Decrease
ENSG00000136929	HEMGN	0.087	9.49E-45	Decrease
ENSG00000128285	MCHR1	0.230	9.66E-45	Decrease
ENSG00000106031	HOXA13	-0.183	2.07E-44	Increase
ENSG00000213417	KRTAP2-4	0.328	2.10E-44	Decrease
ENSG00000116194	ANGPTL1	0.292	2.45E-44	Decrease

ENSG00000181499	OR6T1	0.072	2.64E-44	Decrease
ENSG00000235397	EPN2-AS1	0.402	3.16E-44	Decrease
ENSG00000160472	TMEM190	0.328	3.24E-44	Decrease
ENSG00000228630	HOTAIR	-0.182	4.65E-44	Increase
ENSG00000212724	KRTAP2-3	0.333	6.77E-44	Decrease
ENSG00000105996	HOXA2	-0.308	7.24E-44	Increase
ENSG00000157766	ACAN	0.142	7.50E-44	Decrease
ENSG00000266297	MIR744	0.368	8.00E-44	Decrease
ENSG00000151379	MSGN1	0.090	1.26E-43	Decrease
ENSG00000166682	TMPRSS5	0.198	1.30E-43	Decrease
ENSG00000225626	C9orf135-AS1	-0.125	1.46E-43	Increase
ENSG00000089225	TBX5	-0.225	1.60E-43	Increase
ENSG00000231654	RPS6KA2-AS1	0.370	1.87E-43	Decrease
ENSG00000224729	PCOLCE-AS1	0.288	2.14E-43	Decrease
ENSG00000211517	MIR671	0.207	2.97E-43	Decrease
ENSG00000160360	GPSM1	0.206	3.51E-43	Decrease
ENSG00000119938	PPP1R3C	0.325	4.10E-43	Decrease
ENSG00000163586	FABP1	0.303	4.59E-43	Decrease
ENSG00000164309	CMYA5	0.340	5.29E-43	Decrease
ENSG00000128652	HOXD3	-0.249	7.97E-43	Increase
ENSG00000166446	CDYL2	0.141	8.73E-43	Decrease
ENSG00000182223	ZAR1	0.138	1.18E-42	Decrease
ENSG00000207613	MIR181C	0.150	1.21E-42	Decrease
ENSG00000011465	DCN	0.271	1.21E-42	Decrease
ENSG00000205560	CPT1B	0.235	1.24E-42	Decrease
ENSG00000207639	MIR193B	0.371	1.43E-42	Decrease
ENSG00000170279	C7orf33	0.201	1.48E-42	Decrease
ENSG00000179296	CTGLF12P	0.271	1.53E-42	Decrease
ENSG00000224652	LINC00885	0.250	1.93E-42	Decrease
ENSG00000259218	LINC00928	0.327	1.96E-42	Decrease
ENSG00000174343	CHRNA9	0.150	3.09E-42	Decrease
ENSG00000178934	LGALS7B	0.104	3.11E-42	Decrease
ENSG00000236581	STARD13-AS	0.366	3.64E-42	Decrease
ENSG00000227630	LINC01132	-0.251	3.79E-42	Increase
ENSG00000122863	CHST3	0.218	4.44E-42	Decrease
ENSG00000261279	ULK4P1	0.276	4.68E-42	Decrease
ENSG00000168542	COL3A1	0.366	5.77E-42	Decrease
ENSG00000205899	BHLHA9	0.136	6.86E-42	Decrease
ENSG00000118523	CTGF	0.228	8.32E-42	Decrease
ENSG00000140287	HDC	0.189	9.15E-42	Decrease
ENSG00000269186	LINC01082	0.173	1.18E-41	Decrease
ENSG00000225179	LINC00457	0.278	1.35E-41	Decrease
ENSG00000183643	C15orf32	0.202	1.46E-41	Decrease

ENSG00000165138	ANKS6	0.208	1.46E-41	Decrease
ENSG00000224405	LINC00572	0.241	1.46E-41	Decrease
ENSG00000147613	PSKH2	0.173	1.46E-41	Decrease
ENSG00000202529	SNORD18B	0.260	1.74E-41	Decrease
ENSG00000223843	EFCAB6-AS1	0.189	1.77E-41	Decrease
ENSG00000160307	S100B	0.258	1.82E-41	Decrease
ENSG00000137273	FOXF2	-0.196	2.05E-41	Increase
ENSG00000203618	GP1BB	-0.379	2.66E-41	Increase
ENSG00000168676	KCTD19	0.289	2.73E-41	Decrease
ENSG00000137441	FGFBP2	0.208	3.31E-41	Decrease
ENSG00000214518	KRTAP2-2	0.152	3.95E-41	Decrease
ENSG00000208017	MIR140	0.471	5.14E-41	Decrease
ENSG00000152049	KCNE4	0.268	7.36E-41	Decrease
ENSG00000196666	FAM180B	0.234	7.64E-41	Decrease
ENSG00000240194	CYMP	0.199	7.77E-41	Decrease
ENSG00000114993	RTKN	0.205	8.56E-41	Decrease
ENSG00000120094	HOXB1	0.314	8.73E-41	Decrease
ENSG00000202569	MIR146B	0.264	8.89E-41	Decrease
ENSG00000248593	DSTNP2	0.186	9.03E-41	Decrease
ENSG00000056736	IL17RB	0.099	9.10E-41	Decrease
ENSG00000203801	LINC00222	0.256	1.00E-40	Decrease
ENSG00000198914	POU3F3	-0.139	1.15E-40	Increase
ENSG00000105519	CAPS	0.268	1.31E-40	Decrease
ENSG00000106689	LHX2	-0.184	1.37E-40	Increase
ENSG00000188662	HILS1	0.230	1.82E-40	Decrease
ENSG00000170458	CD14	0.114	2.01E-40	Decrease
ENSG00000204365	C10orf126	0.211	2.20E-40	Decrease
ENSG00000115325	DOK1	0.249	2.50E-40	Decrease
ENSG00000188015	S100A3	0.325	2.50E-40	Decrease
ENSG00000235049	LINC00940	0.139	2.71E-40	Decrease
ENSG00000179058	C9orf50	0.162	2.89E-40	Decrease
ENSG00000197249	SERPINA1	0.175	3.08E-40	Decrease
ENSG00000156966	B3GNT7	0.166	4.73E-40	Decrease
ENSG00000267519	MIR24-2	0.274	5.46E-40	Decrease
ENSG00000204248	COL11A2	0.196	6.66E-40	Decrease
ENSG00000212864	RNF208	0.175	7.03E-40	Decrease
ENSG00000207579	MIR662	0.165	7.73E-40	Decrease
ENSG00000226968	LINC00423	0.286	1.30E-39	Decrease
ENSG00000148795	CYP17A1	0.124	1.51E-39	Decrease
ENSG00000162366	PDZK1IP1	0.157	1.68E-39	Decrease
ENSG00000122543	OCM	0.180	1.77E-39	Decrease
ENSG00000152093	CFC1B	-0.392	2.65E-39	Increase
ENSG00000100665	SERPINA4	-0.018	2.69E-39	Increase
ENSG00000166741	NNMT	0.268	2.72E-39	Decrease
ENSG00000113140	SPARC	0.200	2.98E-39	Decrease
ENSG00000159495	TGM7	0.149	3.20E-39	Decrease

ENSG00000207964	MIR628	0.319	3.58E-39	Decrease
ENSG00000157150	TIMP4	0.160	4.41E-39	Decrease
ENSG00000121316	PLBD1	0.131	4.97E-39	Decrease
ENSG00000225493	LINC01107	0.169	5.12E-39	Decrease
ENSG00000188649	CC2D2B	0.116	5.32E-39	Decrease
ENSG00000007312	CD79B	-0.044	5.34E-39	Increase
ENSG00000114378	HYAL1	0.270	6.69E-39	Decrease
ENSG00000106258	CYP3A5	0.277	7.28E-39	Decrease
ENSG00000131668	BARX1	-0.177	1.22E-38	Increase
ENSG00000179914	ITLN1	0.175	1.27E-38	Decrease
ENSG00000108244	KRT23	0.044	1.39E-38	Decrease
ENSG00000256453	DND1	0.152	1.54E-38	Decrease
ENSG00000156509	FBXO43	0.065	1.57E-38	Decrease
ENSG00000092607	TBX15	0.106	1.68E-38	Decrease
ENSG00000136457	CHAD	0.198	1.92E-38	Decrease
ENSG00000109113	RAB34	0.158	1.95E-38	Decrease
ENSG00000189367	KIAA0408	0.424	1.96E-38	Decrease
ENSG00000237879	LINC00398	0.201	2.13E-38	Decrease
ENSG00000145794	MEGF10	0.133	2.88E-38	Decrease
ENSG00000165828	PRAP1	0.157	3.36E-38	Decrease
ENSG00000173171	MTX1	0.036	4.21E-38	Decrease
ENSG00000033100	CHPF2	0.108	4.91E-38	Decrease
ENSG00000100078	PLA2G3	0.182	5.18E-38	Decrease
ENSG00000160183	TMPRSS3	0.153	5.67E-38	Decrease
ENSG00000212102	MIR301B	0.241	5.94E-38	Decrease
ENSG00000140044	JDP2	0.215	6.39E-38	Decrease
ENSG00000179044	EXOC3L1	0.121	7.03E-38	Decrease
ENSG00000182600	C2orf82	0.192	7.48E-38	Decrease
ENSG00000101405	OXT	0.197	7.80E-38	Decrease
ENSG00000235285	SMIM2-IT1	0.315	8.03E-38	Decrease
ENSG00000148671	ADIRF	0.161	9.31E-38	Decrease
ENSG00000207948	MIR328	0.046	1.03E-37	Decrease
ENSG00000253293	HOXA10	0.158	1.03E-37	Decrease
ENSG00000168060	NAALADL1	0.127	1.11E-37	Decrease
ENSG00000143512	HHIPL2	0.126	1.13E-37	Decrease
ENSG00000162576	MXRA8	0.146	1.13E-37	Decrease
ENSG00000144115	THNSL2	0.119	1.25E-37	Decrease
ENSG00000160678	S100A1	0.262	1.68E-37	Decrease
ENSG00000134061	CD180	0.180	2.07E-37	Decrease
ENSG00000007171	NOS2	0.117	2.25E-37	Decrease
ENSG00000171812	COL8A2	0.182	2.44E-37	Decrease
ENSG00000125844	RRBP1	0.193	3.12E-37	Decrease
ENSG00000233608	TWIST2	0.322	3.84E-37	Decrease
ENSG00000122641	INHBA	0.173	4.56E-37	Decrease
ENSG00000111199	TRPV4	0.134	4.79E-37	Decrease
ENSG00000146666	LINC00525	0.326	4.94E-37	Decrease

ENSG00000258405	ZNF578	0.136	5.02E-37	Decrease
ENSG00000175646	PRM1	0.051	5.12E-37	Decrease
ENSG00000142871	CYR61	0.244	5.65E-37	Decrease
ENSG00000132182	NUP210	0.066	6.06E-37	Decrease
ENSG00000143387	CTSK	0.224	6.42E-37	Decrease
ENSG00000165799	RNASE7	0.207	8.07E-37	Decrease
ENSG00000109610	SOD3	0.063	8.16E-37	Decrease
ENSG00000205086	C2orf91	-0.180	8.61E-37	Increase
ENSG00000272395	IFNL4	0.203	9.01E-37	Decrease
ENSG00000204446	C9orf170	0.167	9.65E-37	Decrease
ENSG00000132669	RIN2	0.203	1.04E-36	Decrease
ENSG00000233791	LINC01136	0.166	1.10E-36	Decrease
ENSG00000139648	KRT71	0.287	1.12E-36	Decrease
ENSG00000088827	SIGLEC1	0.181	1.13E-36	Decrease
ENSG00000128710	HOXD10	-0.146	1.19E-36	Increase
ENSG00000132464	ENAM	0.149	1.34E-36	Decrease
ENSG00000169583	CLIC3	0.197	1.37E-36	Decrease
ENSG00000112761	WISP3	0.236	1.68E-36	Decrease
ENSG00000127423	AUNIP	-0.006	1.72E-36	Increase
ENSG00000138152	BTBD16	0.181	1.72E-36	Decrease
ENSG00000103241	FOXF1	-0.183	1.77E-36	Increase
ENSG00000204282	TNRC6C-AS1	0.077	2.10E-36	Decrease
ENSG00000166917	MIR202HG	0.188	2.38E-36	Decrease
ENSG00000167968	DNASE1L2	0.141	2.99E-36	Decrease
ENSG00000167914	GSDMA	0.095	3.08E-36	Decrease
ENSG00000175664	TEX26	0.115	3.40E-36	Decrease
ENSG00000183036	PCP4	0.115	3.42E-36	Decrease
ENSG00000079101	CLUL1	0.207	3.58E-36	Decrease
ENSG00000183798	EMILIN3	0.110	3.81E-36	Decrease
ENSG00000177363	LRRN4CL	0.248	3.87E-36	Decrease
ENSG00000183186	C2CD4C	0.119	4.15E-36	Decrease
ENSG00000144677	CTDSPL	0.143	4.23E-36	Decrease
ENSG00000188032	C19orf67	0.179	5.20E-36	Decrease
ENSG00000267532	MIR497HG	0.224	5.33E-36	Decrease
ENSG00000164283	ESM1	0.179	5.50E-36	Decrease
ENSG00000118702	GHRH	0.130	5.55E-36	Decrease
ENSG00000204414	CSHL1	0.126	6.54E-36	Decrease
ENSG00000171403	KRT9	0.140	7.00E-36	Decrease
ENSG00000167194	C16orf92	0.134	7.35E-36	Decrease
ENSG00000247095	MIR210HG	0.146	7.35E-36	Decrease
ENSG00000185955	C7orf61	0.041	7.49E-36	Decrease
ENSG00000163975	MFI2	0.157	7.62E-36	Decrease
ENSG00000196878	LAMB3	0.149	8.23E-36	Decrease
ENSG00000211581	MIR765	0.111	8.31E-36	Decrease
ENSG00000204511	MCCD1	0.189	9.23E-36	Decrease

ENSG00000188277	C15orf62	0.259	9.84E-36	Decrease
ENSG00000164106	SCRG1	0.188	1.08E-35	Decrease
ENSG00000213937	CLDN9	0.196	1.10E-35	Decrease
ENSG00000260455	CASC14	-0.255	1.10E-35	Increase
ENSG00000187492	CDHR4	0.048	1.20E-35	Decrease
ENSG00000213199	ASIC3	0.117	1.28E-35	Decrease
ENSG00000172037	LAMB2	0.155	1.32E-35	Decrease
ENSG00000137285	TUBB2B	-0.111	1.35E-35	Increase
ENSG00000237523	LINC00857	0.277	1.88E-35	Decrease
ENSG00000109846	CRYAB	0.165	1.93E-35	Decrease
ENSG00000142632	ARHGEF19	0.153	2.09E-35	Decrease
ENSG00000080166	DCT	0.169	2.36E-35	Decrease
ENSG00000182035	ADIG	0.178	2.50E-35	Decrease
ENSG00000197757	HOXC6	-0.108	2.57E-35	Increase
ENSG00000110852	CLEC2B	-0.157	2.82E-35	Increase
ENSG00000166250	CLMP	0.134	3.23E-35	Decrease
ENSG00000119699	TGFB3	0.227	3.65E-35	Decrease
ENSG00000177096	FAM109B	0.142	3.85E-35	Decrease
ENSG00000142182	DNMT3L	0.074	5.51E-35	Decrease
ENSG00000125089	SH3TC1	0.183	5.89E-35	Decrease
ENSG00000131095	GFAP	0.090	6.57E-35	Decrease
ENSG00000154175	ABI3BP	0.157	6.80E-35	Decrease
ENSG00000137673	MMP7	0.117	6.89E-35	Decrease
ENSG00000115556	PLCD4	0.156	7.43E-35	Decrease
ENSG00000196091	MYBPC1	0.137	8.61E-35	Decrease
ENSG00000113578	FGF1	0.170	8.61E-35	Decrease
ENSG00000140575	IQGAP1	0.165	9.39E-35	Decrease
ENSG00000182107	TMEM30B	0.202	1.05E-34	Decrease
ENSG00000199038	MIR210	0.079	1.08E-34	Decrease
ENSG00000166272	WBP1L	0.176	1.12E-34	Decrease
ENSG00000137142	IGFBPL1	0.140	1.13E-34	Decrease
ENSG00000134198	TSPAN2	0.168	1.16E-34	Decrease
ENSG00000132481	TRIM47	0.101	1.20E-34	Decrease
ENSG00000126562	WNK4	-0.122	1.39E-34	Increase
ENSG00000197587	DMBX1	0.097	1.53E-34	Decrease
ENSG00000005513	SOX8	0.179	1.55E-34	Decrease
ENSG00000188782	CATSPER4	0.148	1.61E-34	Decrease
ENSG00000254417	ANO1-AS2	0.225	1.81E-34	Decrease
ENSG00000215187	FAM166B	0.211	1.87E-34	Decrease
ENSG00000119147	C2orf40	0.096	1.97E-34	Decrease
ENSG00000115318	LOXL3	0.199	2.05E-34	Decrease
ENSG00000198542	ITGBL1	0.155	2.53E-34	Decrease
ENSG00000124610	HIST1H1A	0.146	2.66E-34	Decrease
ENSG00000204928	GRXCR2	0.075	2.71E-34	Decrease
ENSG00000135902	CHRND	0.127	2.71E-34	Decrease
ENSG00000196739	COL27A1	0.139	2.76E-34	Decrease

ENSG00000137801	THBS1	0.153	2.79E-34	Decrease
ENSG00000180638	SLC47A2	0.145	2.93E-34	Decrease
ENSG00000189129	PLAC9	0.138	3.05E-34	Decrease
ENSG00000244242	IFITM10	0.203	3.10E-34	Decrease
ENSG00000123201	GUCY1B2	0.081	3.32E-34	Decrease
ENSG00000248323	LUCAT1	0.111	3.35E-34	Decrease
ENSG00000224116	INHBA-AS1	0.160	3.51E-34	Decrease
ENSG00000182397	DNM1P46	0.029	3.57E-34	Decrease
ENSG00000241839	PLEKHO2	0.204	3.62E-34	Decrease
ENSG00000019102	VSIG2	0.211	3.84E-34	Decrease
ENSG00000178803	ADORA2A-AS1	0.129	4.29E-34	Decrease
ENSG00000187066	TMEM262	0.092	4.37E-34	Decrease
ENSG00000106004	HOXA5	-0.230	4.44E-34	Increase
ENSG00000196966	HIST1H3E	0.262	4.58E-34	Decrease
ENSG00000224057	EGFR-AS1	-0.092	4.80E-34	Increase
ENSG00000151006	PRSS53	0.129	4.86E-34	Decrease
ENSG00000169903	TM4SF4	0.033	5.66E-34	Decrease
ENSG00000121068	TBX2	-0.195	5.95E-34	Increase
ENSG00000169495	HTRA4	0.205	6.07E-34	Decrease
ENSG00000132688	NES	0.111	6.21E-34	Decrease
ENSG00000122862	SRGN	0.115	6.66E-34	Decrease
ENSG00000207719	MIR623	-0.337	6.82E-34	Increase
ENSG00000127325	BEST3	0.190	7.12E-34	Decrease
ENSG00000186897	C1QL4	-0.011	7.20E-34	Increase
ENSG00000214597	TMEM249	0.090	7.23E-34	Decrease
ENSG00000197245	FAM110D	0.127	7.47E-34	Decrease
ENSG00000111145	ELK3	0.164	8.22E-34	Decrease
ENSG00000101276	SLC52A3	0.106	8.41E-34	Decrease
ENSG00000243543	WFDC6	0.232	9.18E-34	Decrease
ENSG00000134107	BHLHE40	0.202	9.92E-34	Decrease
ENSG00000166863	TAC3	0.138	1.01E-33	Decrease
ENSG00000106341	PPP1R17	-0.119	1.16E-33	Increase
ENSG00000137634	NXPE4	0.174	1.21E-33	Decrease
ENSG00000006377	DLX6	-0.106	1.24E-33	Increase
ENSG00000130529	TRPM4	0.149	1.26E-33	Decrease
ENSG00000198523	PLN	-0.339	1.38E-33	Increase
ENSG00000099260	PALMD	0.118	1.40E-33	Decrease
ENSG00000112414	GPR126	0.089	1.41E-33	Decrease
ENSG00000101236	RNF24	0.191	1.41E-33	Decrease
ENSG00000154736	ADAMTS5	0.149	1.43E-33	Decrease
ENSG00000172349	IL16	0.095	1.51E-33	Decrease
ENSG00000163565	IFI16	-0.227	1.51E-33	Increase
ENSG00000090534	THPO	0.095	1.70E-33	Decrease
ENSG00000183397	C19orf71	0.171	1.79E-33	Decrease
ENSG00000127578	WFIKKN1	0.107	2.17E-33	Decrease

ENSG00000141756	FKBP10	0.115	2.25E-33	Decrease
ENSG00000232258	TMEM114	0.181	2.50E-33	Decrease
ENSG00000053702	NRIP2	0.151	2.69E-33	Decrease
ENSG00000146859	TMEM140	-0.170	3.24E-33	Increase
ENSG00000101000	PROCR	0.151	3.29E-33	Decrease
ENSG00000254008	LINC00051	0.204	3.42E-33	Decrease
ENSG00000143032	BARHL2	0.070	3.47E-33	Decrease
ENSG00000250251	PKD1P6	0.206	3.60E-33	Decrease
ENSG00000237424	FOXD2-AS1	-0.126	3.69E-33	Increase
ENSG00000148677	ANKRD1	0.209	3.82E-33	Decrease
ENSG00000212899	KRTAP3-3	0.160	3.97E-33	Decrease
ENSG00000189134	NKAPL	0.228	4.07E-33	Decrease
ENSG00000108679	LGALS3BP	-0.021	4.38E-33	Increase
ENSG00000144063	MALL	0.176	4.62E-33	Decrease
ENSG00000125650	PSPN	0.120	4.69E-33	Decrease
ENSG00000131480	AOC2	0.026	5.28E-33	Decrease
ENSG00000019991	HGF	0.061	5.81E-33	Decrease
ENSG00000061455	PRDM6	-0.164	5.88E-33	Increase
ENSG00000164920	OSR2	-0.093	6.09E-33	Increase
ENSG00000257859	CASC18	0.184	6.98E-33	Decrease
ENSG00000199075	MIR26A1	0.173	7.33E-33	Decrease
ENSG00000183072	NKX2-5	-0.145	7.40E-33	Increase
ENSG00000232709	MARK2P9	0.110	7.85E-33	Decrease

Abbreviations: OA osteoarthritis

Supplementary Table S4. The list of the 66 DMGs between pancreatic β-cells and other tissues based on FDR.

Ensembl id	Gene symbol	Mean methylation difference	FDR	Methylation in pancreatic β-cells in relation to other tissues
ENSG00000215695	RSC1A1	-0.692	9.72E-04	Decrease
ENSG00000199025	MIR369	-0.149	8.35E-05	Decrease
ENSG00000199012	MIR412	-0.343	9.37E-05	Decrease
ENSG00000251791	SCARNA6	-0.561	1.22E-04	Decrease
ENSG00000171133	OR2K2	-0.529	1.91E-04	Decrease
ENSG00000254647	INS	-0.512	8.81E-04	Decrease
ENSG00000207588	MIR593	-0.631	1.35E-03	Decrease
ENSG00000198271	KRTAP4-5	-0.620	1.35E-03	Decrease
ENSG00000221036	MIR1193	-0.523	1.90E-03	Decrease
ENSG00000163497	FEV	0.260	1.94E-03	Increase
ENSG00000117242	PINK1-AS	-0.516	1.94E-03	Decrease
ENSG00000126266	FFAR1	-0.546	2.43E-03	Decrease
ENSG00000167615	LENG8	-0.332	3.07E-03	Decrease
ENSG00000150594	ADRA2A	-0.254	3.75E-03	Decrease
ENSG00000124701	APOBEC2	-0.393	4.49E-03	Decrease
ENSG00000019505	SYT13	-0.345	4.75E-03	Decrease
ENSG00000143178	TBX19	-0.199	5.47E-03	Decrease

ENSG00000211582	MIR758	-0.410	6.82E-03	Decrease
ENSG00000100253	MIOX	-0.363	7.99E-03	Decrease
ENSG00000208036	MIR106B	-0.567	8.40E-03	Decrease
ENSG00000197763	TXNRD3	-0.241	8.40E-03	Decrease
ENSG00000167230	C17orf78	-0.256	8.49E-03	Decrease
ENSG00000119487	MAPKAP1	-0.192	9.55E-03	Decrease
ENSG00000231322	RPL13AP17	-0.623	9.96E-03	Decrease
ENSG00000182271	TMIGD1	-0.591	1.12E-02	Decrease
ENSG00000148291	SURF2	-0.236	1.12E-02	Decrease
ENSG00000214262	ANKRD36BP1	-0.552	1.15E-02	Decrease
ENSG00000205882	DEFB134	-0.458	1.15E-02	Decrease
ENSG00000207696	MIR659	-0.400	1.15E-02	Decrease
ENSG00000266239	MIR3605	-0.112	1.15E-02	Decrease
ENSG00000251372	LINC00499	-0.600	1.34E-02	Decrease
ENSG00000207630	MIR7-3	-0.467	1.48E-02	Decrease
ENSG00000116786	PLEKHM2	-0.181	1.79E-02	Decrease
ENSG00000254934	LINC00678	-0.482	1.97E-02	Decrease
ENSG00000252835	SCARNA21	-0.221	1.97E-02	Decrease
ENSG00000184933	OR6A2	-0.449	1.98E-02	Decrease
ENSG00000249553	PPP2R2B-IT1	-0.302	1.98E-02	Decrease
ENSG00000014164	ZC3H3	-0.265	2.47E-02	Decrease
ENSG00000234854	LINC00676	-0.689	2.77E-02	Decrease
ENSG00000209702	SNORD41	-0.375	2.87E-02	Decrease
ENSG00000238531	SNORD105B	-0.347	3.03E-02	Decrease
ENSG00000176555	OR4S1	-0.298	3.06E-02	Decrease
ENSG00000107262	BAG1	-0.339	3.33E-02	Decrease
ENSG00000207978	MIR154	-0.341	3.41E-02	Decrease
ENSG00000201785	SNORD117	-0.611	3.57E-02	Decrease
ENSG00000168067	MAP4K2	-0.266	3.63E-02	Decrease
ENSG00000207948	MIR328	-0.131	3.66E-02	Decrease
ENSG00000134489	HRH4	-0.082	3.66E-02	Decrease
ENSG00000212040	MIR543	-0.321	3.75E-02	Decrease
ENSG00000149634	SPATA25	-0.120	3.75E-02	Decrease
ENSG00000212443	SNORA53	-0.546	3.84E-02	Decrease
ENSG00000238961	SNORA47	-0.426	3.94E-02	Decrease
ENSG00000207562	MIR34C	-0.355	4.10E-02	Decrease
ENSG00000170279	C7orf33	-0.286	4.44E-02	Decrease
ENSG00000157106	SMG1	-0.252	4.44E-02	Decrease
ENSG00000157999	ANKRD61	-0.169	4.44E-02	Decrease
ENSG00000127366	TAS2R5	-0.114	4.44E-02	Decrease
ENSG00000205856	C22orf42	-0.482	4.51E-02	Decrease
ENSG00000165188	RNF183	-0.321	4.51E-02	Decrease
ENSG00000212498	SNORD86	-0.110	4.57E-02	Decrease
ENSG00000167173	C15orf39	-0.178	4.58E-02	Decrease
ENSG00000201772	SNORA5C	-0.167	4.60E-02	Decrease
ENSG00000253799	LINC01030	-0.646	4.65E-02	Decrease

ENSG00000213339	QTRT1	-0.252	4.96E-02	Decrease
